# Cutaneous HPV8 and MmuPV1 E6 Proteins Target the NOTCH and TGF-β Tumor Suppressors to Inhibit Differentiation and Sustain Keratinocyte Proliferation

**DOI:** 10.1371/journal.ppat.1006171

**Published:** 2017-01-20

**Authors:** Jordan M. Meyers, Aayushi Uberoi, Miranda Grace, Paul F. Lambert, Karl Munger

**Affiliations:** 1 Program in Virology, Harvard Medical School, Boston, Massachusetts, United States of America; 2 Department of Developmental, Molecular and Chemical Biology, Tufts University School of Medicine, Boston, Massachusetts, United States of America; 3 McArdle Laboratory for Cancer Research, Department of Oncology, School of Medicine and Public Health, University of Wisconsin-Madison, Madison, Wisconsin, United States of America; Fred Hutchinson Cancer Research Center, UNITED STATES

## Abstract

Cutaneous beta-papillomaviruses are associated with non-melanoma skin cancers that arise in patients who suffer from a rare genetic disorder, Epidermodysplasia verruciformis (EV) or after immunosuppression following organ transplantation. Recent studies have shown that the E6 proteins of the cancer associated beta human papillomavirus (HPV) 5 and HPV8 inhibit NOTCH and TGF-β signaling. However, it is unclear whether disruption of these pathways may contribute to cutaneous HPV pathogenesis and carcinogenesis. A recently identified papillomavirus, MmuPV1, infects laboratory mouse strains and causes cutaneous skin warts that can progress to squamous cell carcinoma. To determine whether MmuPV1 may be an appropriate model to mechanistically dissect the molecular contributions of cutaneous HPV infections to skin carcinogenesis, we investigated whether MmuPV1 E6 shares biological and biochemical activities with HPV8 E6. We report that the HPV8 and MmuPV1 E6 proteins share the ability to bind to the MAML1 and SMAD2/SMAD3 transcriptional cofactors of NOTCH and TGF-beta signaling, respectively. Moreover, we demonstrate that these cutaneous papillomavirus E6 proteins inhibit these two tumor suppressor pathways and that this ability is linked to delayed differentiation and sustained proliferation of differentiating keratinocytes. Furthermore, we demonstrate that the ability of MmuPV1 E6 to bind MAML1 is necessary for papilloma formation in experimentally infected mice. Our results, therefore, suggest that experimental MmuPV1 infection in mice will be a robust and useful experimental system to model key aspects of cutaneous HPV infection, pathogenesis and carcinogenesis.

## Author Summary

The lack of a genetically tractable small animal model to study viral infection and pathogenesis has significantly hindered papillomavirus research. The recent discovery of Mus musculus papillomavirus 1 (MmuPV1), which can replicate and form skin warts and cancers in experimentally infected laboratory mouse strains, has the potential to be transformative to the field. However, it is important to determine whether MmuPV1 targets some of the same cellular signaling pathways as the skin cancer-associated human papillomaviruses (HPVs). We show that MmuPV1 E6 shares with the skin cancer-associated HPV8 E6 protein the capacity to inhibit NOTCH and TGF-β signaling. Importantly, MmuPV1 E6 expression and specifically the ability of E6 to inhibit NOTCH signaling are necessary for wart formation in mice. Hence, MmuPV1 will be an excellent animal model to study key aspects of the life cycle and pathogenesis of skin cancer-associated HPVs.

## Introduction

Papillomaviruses (PVs) represent a large, diverse group of DNA viruses that infect squamous epithelia of many animals. Genetically, these viruses are grouped into genera based on diversity of their major capsid protein. Biologically, PVs can be stratified according to the type of epithelium that they can productively infect, either mucosal or cutaneous tissue. Infections with cutaneous human PVs (HPVs) is associated with a wide range of pathologies from asymptomatic infection to benign warts, actinic keratosis to squamous cell carcinomas [1]. Two HPV types in particular, beta-HPV5 and beta-HPV8, were found to be associated with lesions and tumors in patients suffering from a rare hereditary disease, epidermodysplasia verruciformis (EV) [2]. A majority of these patients harbor genetic mutations in TMC6 (EVER1) or TMC8 (EVER2) genes on chromosome 17, which encode putative transmembrane channel proteins that may be involved in cellular zinc and calcium homeostasis [3, 4]. HPV-associated warts in EV patients have a high risk for progression to squamous cell carcinomas (SCC), and these tumors are positive for viral DNA [5]. Further studies provided evidence that SCCs that arise in immunosuppressed individuals such as organ transplant patients are also associated with cutaneous HPV infections [6, 7]. HPV-associated SCCs often arise in sun-exposed areas of the skin, implicating UV exposure as a key risk factor for malignant progression [8]. However, beta-HPV sequences are not maintained in every cancer cell. The association of cutaneous HPV infections with SCC in immunocompetent patients is less clear. One study showed a positive serological connection between HPV8 and SCC [9], but other studies have not shown a link [10]. This has led to a gradated model of beta-HPV association with human skin cancers. Cancer development is highly associated in the case of EV patients, correlated in the case of immune suppression, and only slightly or sporadically associated in immune competent patients. However, in transgenic mouse models skin restricted expression of the beta-HPV oncogenes E6 and E7 are capable of tumorigenesis suggesting that oncogene expression may play an important role [11–13].

In order to assess HPV contributions to skin carcinogenesis, it is important to define the effects of cutaneous HPVs on host cell pathways. UV exposure is an important risk factor for skin cancer, and several reports suggest that cells expressing cutaneous HPV E6 proteins can tolerate or survive UV exposure and UV-induced DNA damage better than normal cells. It has been shown that HPV5 E6 can inhibit apoptotic cell death in response to UV through degradation of the pro-apoptotic BCL2 family member, BAK1 [14, 15]. Moreover, activation of the ATM/ATR kinases that play an important role in UV induced DNA damage signaling is also inhibited in cutaneous HPV E6 expressing keratinocytes, and this has been linked to E6 mediated EP300/CREBBP degradation [16, 17].

Beyond modulation of the cellular UV response, cutaneous HPVs have additional oncogenic activities, and our group along with several others discovered that HPV8 E6 can bind to MAML1 and inhibit NOTCH signaling [18–20]. NOTCH is an important driver of keratinocyte differentiation, and defects in the pathway are highly associated with cutaneous SCCs [21, 22]. Additionally, HPV5 E6 was shown to interact with SMAD3 causing its destabilization and subsequently inhibiting TGF-β signaling [23]. SMAD2 and SMAD3 are TGF-β receptor associated factors that are activated by phosphorylation upon TGF-β1 ligand binding and translocate to the nucleus. They form complexes with non-receptor associated SMADs and transcriptional co-activators to bind and activate expression of TGF-β target genes. TGF-β also plays an important, albeit complicated role in skin carcinogenesis, as it has both tumor suppressive and tumor promoting activities; loss of cytostatic TGF-β signaling is important during the early phase of carcinogenic progression, whereas TGF-β signaling may drive late stage carcinogenic events including invasion and metastasis through activation of the epithelial to mesenchymal transition (EMT) [24, 25].

In addition to understanding the effects of beta-HPV oncogenes on host pathways, it would be beneficial to have a robust model system to study viral pathogenesis and oncogenesis. One principal hurdle in investigating HPV pathogenesis and oncogenesis has been the difficulty to study these viruses in appropriate experimental model systems. The exquisite host specificity of papillomaviruses has precluded experimental infections of heterologous hosts and the viral life cycle cannot be fully studied in conventional tissue culture experiments. Organotypic “raft” culture systems have been developed to study aspects of the productive life cycle of high-risk mucosotropic HPVs [26], but cutaneous HPVs such as HPV5 and HPV8 have not been studied in this system. Moreover, raft cultures do not faithfully recapitulate the steady state physiology of the skin, and thus they cannot be used to examine long-term persistent HPV infections. To circumvent some of these issues, researchers has resorted to growing HPV genome expressing human keratinocytes in implantation chambers on the backs of immunodeficient mice [27] or culturing them in the renal capsule [28]. The recent isolation of a mouse PV, MmuPV1, from warts developed in a colony of NMRI-Foxn1^nu^/Foxn1^nu^ (nude) mice [29] has provided an important breakthrough and now allows PV pathogenesis studies in a genetically tractable animal model. Infectious MmuPV1 quasivirions that are synthesized *in vitro* can be used for experimental infections that result in warts [30]. Alternatively, circularized viral genomes directly applied to scarified skin regions also lead to wart formation [31]. Recent reports have shown that lesions arising due to MmuPV1 infection have malignant potential and in some cases, can progress to SCCs [31]. Moreover, experimental MmuPV1 infection causes papillomas associated with SCC in UVB-irradiated immunocompetent strains of mice. Importantly, these studies showed that MmuPV1 mediated tumor formation was a consequence of UV-induced immunosuppression [32]. Given these biological similarities to cutaneous HPVs, there is an exciting probability that MmuPV1 is a biologically relevant animal papillomavirus model that will be useful in determining whether and how cutaneous HPVs mechanistically contribute to skin carcinogenesis. Therefore, we hypothesized that MmuPV1 gene products must share biochemical properties and biological activities with those of cutaneous HPV gene products. The goal of this study was to identify host cell signal transduction pathways targeted by the MmuPV1 E6 protein, to compare and contrast them with those targeted by HPV8 E6, to determine their effects on proliferation and survival of terminally differentiated keratinocytes, and to assess their role in wart formation in mouse infection models.

## Results

### HPV8 E6 and MmuPV1 share the ability to bind MAML1 and SMAD2/3

**Fig 1:**
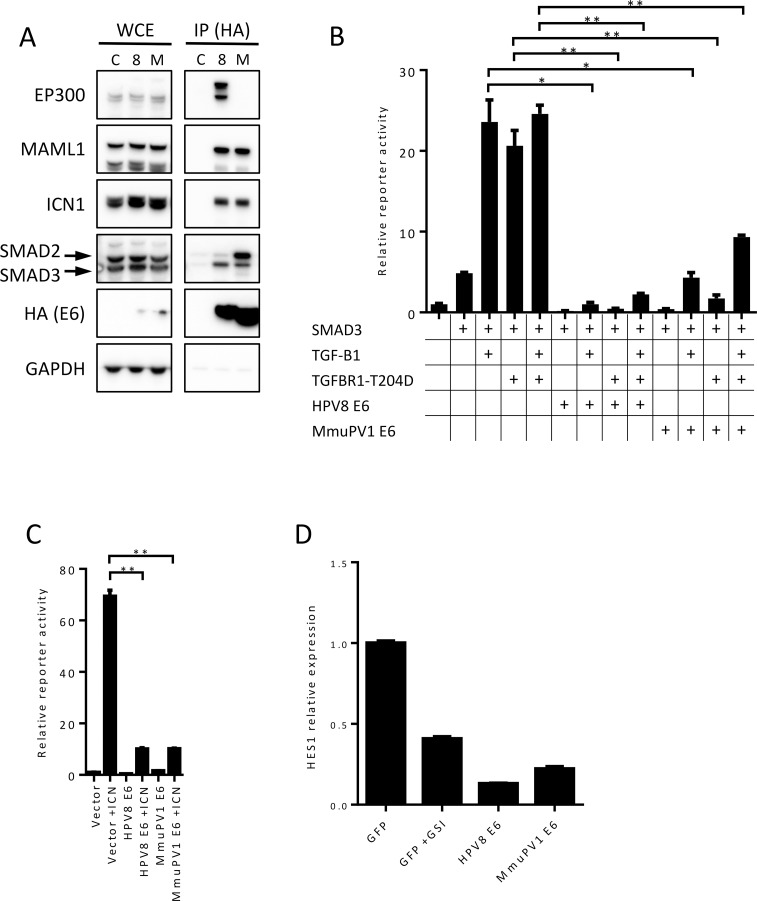
HPV8 and MmuPV1 E6 interact with components of NOTCH and TGF-β signaling pathway and inhibit these pathways. **(A)** Whole cell extracts (WCE) from human keratinocytes stably expressing FLAG/HA-tagged HPV8 E6 (8), MmuPV1 E6 (M), or GFP (C) were subjected to HA immunoprecipitation (IP) and analyzed for associated EP300, MAML1 and ICN1 (three components of NOTCH transcriptional complex) and SMAD2 and SMAD3 (receptor SMADs of TGF-β signaling) as well as immunoprecipitated E6 by immunoblot. Expression of the various proteins was assessed by immunoblotting of 1% input of WCE. GAPDH expression is shown as a loading control. **(B)** Effects of HPV8 and MmuPV1 E6 on SMAD responsive reporter activity using either TGF-β1 (10 ng/ml) or constitutively active TGF-β Receptor 1 (TGFBR1-T204D) as agonists in U2OS cells. **(C)** Effects of HPV8 and MmuPV1 E6 on NOTCH responsive reporter activity using ICN1 as an agonist in U2OS cells. Data shown is from a representative experiment from three independent experiments. P-values were calculated with unpaired t-test with Welch’s correction; * = P ≤ 0.05, ** = P ≤ 0.01. **(D)** Expression level of HES1 transcripts in GFP-control, GSI treated GFP-control, HPV8 E6, and MmuPV1 E6 expressing tert-immortalized human foreskin keratinocytes (iHFKs) as measured by RT-qPCR.

MmuPV1 infections cause skin warts [29–31] that can progress to cancers in conjunction with UV irradiation [32]. To determine whether MmuPV1 may be used to model pathogenesis and carcinogenesis of human cutaneous HPVs, we investigated whether it targets similar cellular signaling pathways as cutaneous HPV5 and 8. We focused on the MmuPV1 E6 protein, because there are marked differences between the protein interactomes of mucosal versus cutaneous HPV E6 proteins [33, 34]. Mucosal HPV16 E6 proteins interact with the LXXLL (L, leucine; X any amino acid) domain containing ubiquitin ligase UBE3A, the TP53 tumor suppressor, and cellular proteins containing a PDZ (post synaptic density protein-PSD95, Drosophila disc large tumor suppressor-Dlg1, and zonula occludens-1 protein-zo-1) domain [33, 35, 36]. In contrast, cutaneous HPV5 and HPV8 E6 proteins interact with the LXXLL domain protein, MAML1, as well as SMAD3, which lacks an LXXLL motif, and they do not bind to PDZ domain proteins because these E6 proteins lack the appropriate C-terminal binding site [19, 20, 23, 35, 37]. To determine whether MmuPV1 E6 interacts with cutaneous HPV5 and HPV8 specific cellular interactors, we infected normal human oral keratinocytes (NOKs) with lentiviral vectors expressing MmuPV1 FLAG/HA-E6, HPV8 FLAG/HA-E6 as a positive control, or GFP as a negative control. Lysates from cell populations with stable expression of the corresponding epitope tagged E6 proteins or GFP were then subject to HA immunoprecipitation followed by immunoblot. These experiments show that similar what we previously observed with HPV8 in immortalized foreskin keratinocytes, HPV8 and the MmuPV1 E6 protein interact with MAML1 as well as with intracellular cleaved NOTCH1 (ICN1) (Fig 1A). Like HPV8 E6, MmuPV1 E6 binds SMAD2 and SMAD3. MmuPV1 E6 preferentially interacts with SMAD2 whereas HPV8 E6 preferentially interacts with SMAD3. Unlike what had been observed with HPV5 E6[23], we did not observe any differences in steady state levels of SMAD2 or SMAD3 in HPV8 E6 or MmuPV1 E6 expressing cells. MmuPV1 E6, unlike HPV8 E6, does not detectably interact with EP300/CREBBP. These results suggest that MmuPV1 E6 does not modulate EP300 activities as has been reported for HPV8 E6 [17, 38, 39] but that MmuPV1 and HPV8 E6 share the capacity to associate with components of the NOTCH and TGF-β tumor suppressor pathways.

### HPV8 and MmuPV1 E6 share the ability to inhibit NOTCH and TGF-β transcriptional activity

It was previously shown that HPV8 can inhibit NOTCH signaling [18–20, 35] and the highly-related HPV5 E6 protein was shown to inhibit TGF-β signaling [23]. When active, both of these pathways are known tumor suppressors in the skin [22, 25]. Hence, we determined whether MmuPV1 E6 inhibited these two tumor suppressor pathways. To assess the effect of HPV8 and MmuPV1 E6 on TGF-β signaling, we performed luciferase assays in U2OS cells using a SMAD responsive luciferase reporter that can monitor transcriptional activity of SMAD2 and SMAD3 after TGF-β stimulation. HPV8 E6 and MmuPV1 E6 expression vectors or empty vector were cotransfected with a SMAD3 expression plasmid. Signaling was activated by adding exogenous TGF-β1, co-transfection of a vector expressing a constitutively active mutant of the TGF-βreceptor 1 (TGFBR1-T204D), or both. Both TGF-β1 treatment and expression of TGFBR1-T204D led to greater than 20-fold increases (23.6±2.7, 20.6±1.9, respectively) in reporter activity as compared to control transfected cells (Fig 1B). Co-transfection of HPV8 or MmuPV1 E6 expression plasmids significantly inhibited the ability to activate this response. To rule out any effects of epitope tags, N-terminally-tagged and untagged constructs of HPV8 E6 and MmuPV1 E6 were assessed side by side and no difference was observed in their ability to inhibit NOTCH and TGF-β reporter induction (S1 Fig). Additionally, we repeated these reporter experiments in TERT immortalized human foreskin keratinocytes (iHFKs) and we obtained similar results as previous obtained with U2OS cells (S2A Fig). These results indicate that although we do not observe destabilization of SMAD2 or SMAD3 (Fig 1A), we do observe inhibition of TGF-β activity by HPV8 E6 and show that cutaneous MmuPV1 E6 shares this ability.

To investigate inhibition of NOTCH signaling we co-transfected a NOTCH responsive luciferase reporter with an expression plasmid encoding the active, cleaved NOTCH fragment (ICN1) and HPV8 E6, MmuPV1 E6 expression vectors, or empty vector into U2OS cells. ICN1 transfection in combination with empty vector yielded a 69.4 (±2.3)-fold increase in NOTCH reporter activity compared to cells transfected with the reporter alone (Fig 1C). Co-transfection of HPV8 E6 inhibited ICN induced reporter activity (10.1±0.4 fold). Co-transfection of MmuPV1 E6 similarly inhibited ICN induced reporter activation (10.15±0.3 fold). Similar to the TGF-beta reporter studies, we tested the effect of HPV8 E6 and MmuPV1 E6 on NOTCH inhibition in iHFKs. Consistent with what we observed with U2OS cells, ICN1 transfection alone resulted in an increase in reporter activity (46.7±2.7 fold), which was inhibited upon HPV8 E6 or MmuPV1 E6 cotransfection (3.6±0.1 and 5.0±0.1 respectively) (S2B Fig). To confirm the ability of HPV8 E6 and MmuPV1 E6 to block expression of direct NOTCH transcriptional targets we assayed the mRNA levels of HES1 a canonical NOTCH regulated target. Similar to the gamma secretase inhibitor compound E (GSI) treated control cells, HPV8 E6, or MmuPV1 E6 expressing cells showed reduced HES1 mRNA compared to untreated control cells. This demonstrates the ability of HPV8 E6 and MmuPV1 E6 to inhibit expression of endogenous NOTCH target genes (Fig 1D). Since ICN1 transfection bypasses regulatory steps at the membrane such as ligand binding and NOTCH cleavage, we conclude that, similar to what we have shown for HPV8 E6, MmuPV1 E6 inhibits NOTCH signaling downstream of early receptor proximal events, presumably through association with MAML1 in the nucleus.

These results demonstrate that MmuPV1 E6 can inhibit NOTCH and TGF-β signaling and that this may a conserved function of skin cancer-associated PVs including HPV5 and HPV8 [19, 20, 23]. We do not observe SMAD destabilization but show evidence that inhibition of these pathways is downstream of ligand binding or other receptor proximal events.

### HPV8 E6 and MmuPV1 E6 abrogate TGF-β induced inhibition of keratinocyte growth

**Fig 2:**
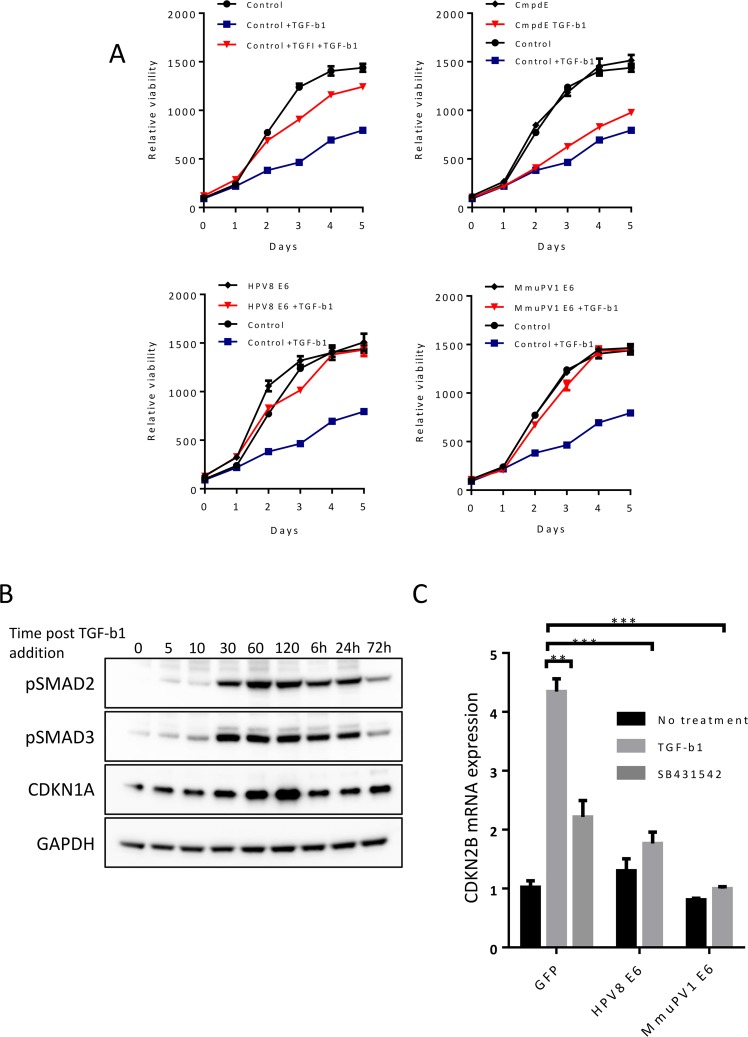
HPV8 and MmuPV1 E6 inhibit TGF-β signaling and activity. **(A)** Proliferation/viability of GFP-control, HPV8 E6, and MmuPV1 E6 expression iHFKs after TGF-β1 (10 ng/ml) treatment over five days as measured by resazurin reduction. P-values (see text) were calculated using Wilcoxon matched-pairs test. **(B)** Accumulation of phosphorylated SMAD2/3 (pSMAD2/3) and expression of CDKN1A (p21^CIP^) following TGF-β1 treatment in iHFKs by immunoblot. **(C)** Expression level of CDKN2B (p15^INK4B^) transcripts in GFP-control, HPV8 E6, and MmuPV1 E6 expressing iHFKs after TGF-β1 treatment as measured by RT-qPCR. P-values were calculated with unpaired t-test with Welch’s correction where * = P ≤ 0.05, ** = P ≤ 0.01, *** = P ≤ 0.001.

TGF-β induces cell-cycle arrest in keratinocytes [40]. Since HPV8 and MmuPV1 E6 can inhibit TGF-β signaling in a reporter assay (Fig 1B), we predicted that HPV8 and MmuPV1 E6 could modify the TGF-β induced growth arrest response in human keratinocytes. We treated iHFKs stably expressing either HPV8 E6 or MmuPV1 E6 or GFP with TGF-β1 (10 ng/ml). Cell proliferation/viability was measured every 24 hours for 5 days using reduction of resazurin, a redox-sensitive dye that interrogates redox fitness of cells, as a proliferation/viability indicator. As expected, control iHFKs treated with TGF-β1 showed a significant decrease in proliferation/viability compared with untreated cells (p-value = 0.0313) (Fig 2A, top left). Concurrent treatment with the TGF-β receptor 1 (TGFBR1) inhibitor SB-431542 (TGFI) abrogates TGF-β growth inhibition (p-value = 0.1563). As expected, treatment with compound E (GSI), a NOTCH inhibitor, did not rescue TGF-β-induced growth arrest (Fig 2A, top right). In contrast, iHFKs expressing either HPV8 E6 (Fig 2A bottom left) or MmuPV1 E6 (Fig 2A, bottom right) are largely resistant to TGF-β1 growth inhibition and proliferate similarly to untreated control iHFKs (p-values = 0.8438 and 0.4375, respectively). These results indicate that, consistent with their capacity to interact with R-SMADs, HPV8 and MmuPV1 E6 render keratinocytes insensitive to TGF-β induced growth arrest (Fig 1A).

### HPV8 E6 and MmuPV1 E6 abrogate TGF-β induced transcriptional responses

Since HPV8 and MmuPV1 E6 expression inhibits reporter assay activity, we hypothesized that expression of these proteins would inhibit endogenous TGF-β responsive transcriptional targets. To test this, we treated GFP expressing iHFKs with TGF-β1 and monitored phosphorylation of SMAD2 and SMAD3 over a time course of 72 hours by immunoblot. Increase of phosphorylation as well as expression of CDKN1A, a TGF-beta target gene, was detected within minutes of treatment and increased to maximal signal by two hours (Fig 2B). Consequently, we analyzed TGF-β transcriptional responses at two hours after TGF-β1 treatment for the remainder of our studies. One important transcriptional target of TGF-β is CDKN2B (p15^INK4B^), a CDK4/CDK6 inhibitor, that blocks G1 progression by inhibiting cyclin D binding. We first verified that CDKN2B expression is dynamically regulated in iHFKs. Cells were treated with TGF-β1, and RNA was harvested two hours after treatment. Expression of CDKN2B was assessed by RT-qPCR. TGF-β1 treatment increased abundance of CDKN2B mRNA in GFP control IHFKs, which was blocked by the (Fig 2C) TGF-β inhibitor SB-431542 (TGFI). Similar to inhibitor treated cells, HPV8 and MmuPV1 E6 iHFKs also failed to induce CDKN2B expression after TGF-β1 treatment. As expected based on our previous reporter assays, HPV8 and MmuPV1 E6 can inhibit the expression of critical TGF-β targets genes following stimulation by TGF-β1.

### HPV8 E6 and MmuPV1 E6 do not interfere with TGF-β mediated SMAD2/3 phosphorylation and nuclear translocation

**Fig 3:**
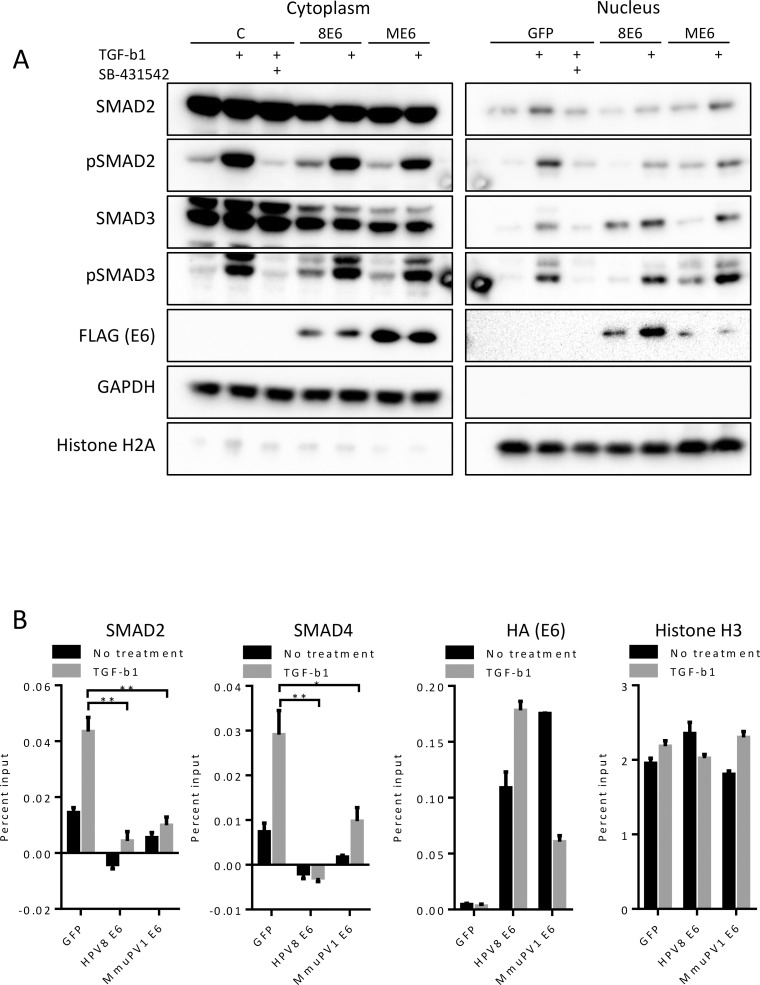
Effects of HPV8 and MmuPV1 E6 on TGF-β nuclear events. **(A)** Immunoblot analysis of TGF-β signaling components following TGF-β1 treatment in cytoplasmic and nuclear fractions of GFP (C), HPV8 E6 (8E6) or MmuPV1 E6 (ME6) expressing iHFKs. Data shown is representative of three independent fractionation experiments. **(B)** Occupancy of SMAD2 and SMAD4 at the TGF-β regulatory site of the CDKN2B gene in GFP-control, HPV8 E6, and MmuPV1 E6 iHFKs as measured by ChIP-qPCR. Data shown is displayed as percent input after subtraction of isotype matched IgG signal. Similar results were obtained from two independent experiments. P-values were calculated with unpaired t-test with Welch’s correction where * = P ≤ 0.05, ** = P ≤ 0.01, *** = P ≤ 0.001.

We sought to better understand the step at which E6 inhibits TGF-β signaling. Normally, receptor-mediated phosphorylation of SMAD2 and SMAD3 leads to their translocation to the nucleus. In the nucleus SMAD2 and SMAD3 complex with SMAD4, a TGF-β coactivator. Contrary to previous studies with HPV5 E6 [23], we did not observe consistent changes to SMAD2 or SMAD3 steady state levels in HPV8 E6 or MmuPV1 E6 expressing iHFKs (Fig 1A). We hypothesized that E6 may block phosphorylation or nuclear translocation of SMADs to inhibit TGF-β signaling. We then examined phosphorylation of SMAD2/3 during TGF-β1 treatment and its subsequent nuclear translocation. To do so, we treated iHFKs with TGF-β1 and harvested the cells 2 hours post treatment, prepared nuclear and cytosolic fractions and performed immunoblot analyses. We observed similar levels of phosphorylated SMAD2 and SMAD3 in the cytosolic and nuclear fractions of HPV8 and MmuPV1 expressing cells as in control iHFKs (Fig 3A). As expected, treatment with the TGF-β inhibitor SB-431542 abrogated phosphorylation and nuclear translocation of SMAD2 and SMAD3. These results indicate that E6 inhibition of TGF-β is downstream of nuclear translocation of phosphorylated SMADs.

### HPV8 E6 and MmuPV1 E6 interfere with TGF-β nuclear complex assembly

Since E6 does not prevent phosphorylated SMADs from entering the nucleus, we predicted it may prevent transcriptional complex formation. After phosphorylation and transport into the nucleus, SMAD2 and SMAD3 proteins form complexes with SMAD4 and assemble at regulatory sites to induce transcription of target genes. The CDKN2B promoter contains a well-defined SMAD binding element (SBE) [41]. We determined whether HPV8 or MmuPV1 E6 disrupt SMAD association with DNA, thereby blocking transcriptional activity. We treated iHFKs with TGF-β1, performed chromatin immunoprecipitations (ChIPs) and measured occupancy of SMAD2 and SMAD4 at the SBE of the CDKN2B promoter. As expected, there was an increase in occupancy of both SMAD2 and SMAD4 after TGF-β1 treatment of control iHFKs (Fig 3B). In contrast, HPV8 E6 and MmuPV1 E6 expressing iHFKs showed reduced SMAD2 and SMAD4 occupancy at the CDKN2B promoter even after TGF-β1 treatment. Surprisingly, HPV8 and MmuPV1 E6 were both detected at the SBE of the CDKN2B promoter. Given that HPV8 and MmuPV1 E6 proteins bind to SMAD2/3 and are not known to directly bind DNA, it is conceivable that E6 binding may interfere with binding of the SMAD2 antibody used for the ChIP assays and occlude the SMAD4 binding site. Another possibility is that E6 may prevent stable SMAD complexes from forming leading to decreased signal of both SMAD2 and SMAD4.

**Fig 4:**
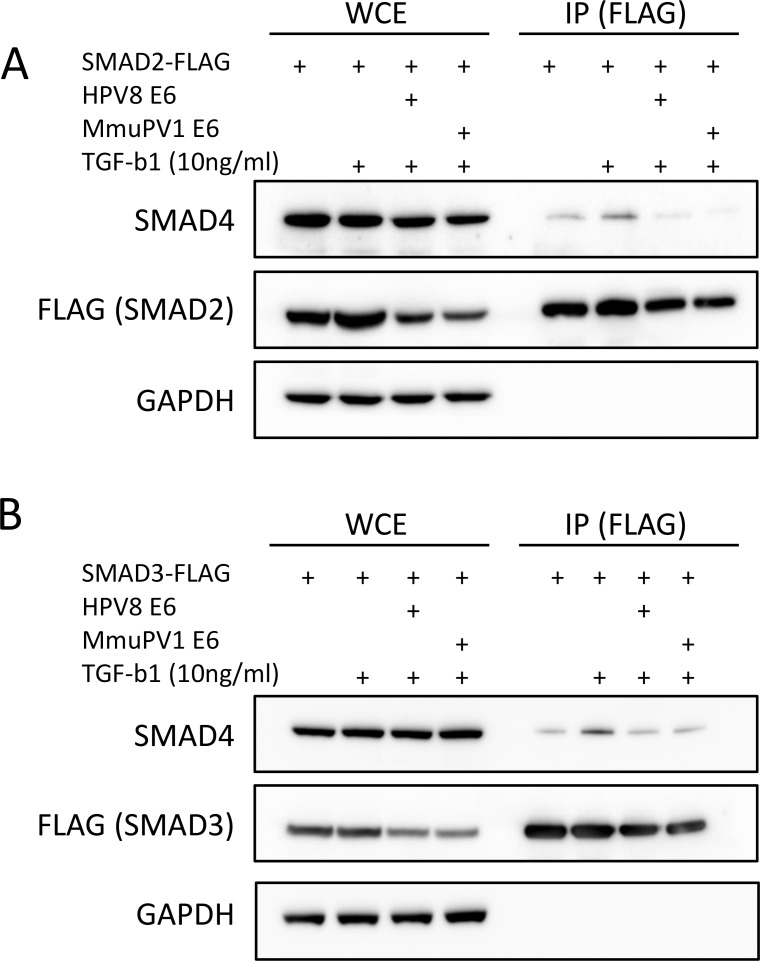
SMAD4 association with SMAD2/3 is abrogated in the presence of HPV8 and MmuPV1 E6. Immunoprecipitation of FLAG-tagged SMAD2 **(A)** or FLAG-tagged SMAD3 **(B)** with co-transfection of either HPV8 E6 or MmupV1 E6 after TGF-β1 treatment followed by SMAD4 immunoblot. Data shown is representative of three independent experiments.

Since we had not been able to observe interaction of either HPV8 E6 or MmuPV1 E6 with SMAD4 by immunoprecipitation (S3 Fig), we hypothesized that one way that E6 may prevent stable SMAD complexes from forming would be that E6 may prevent SMAD4 from interacting with phosphorylated SMAD2/3. To test whether E6 binding to SMAD2/3 precludes SMAD4 binding, we transfected U2OS cells with plasmids encoding a FLAG-tagged SMAD2 and plasmids encoding untagged versions of either HPV8 E6 or MmuPV1 E6. Two days after transfection we treated these cells with TGF-β1 and prepared lysates at two hours after treatment. After immunoprecipitating SMAD2 using FLAG antibodies we analyzed co-precipitated SMAD4 by immunoblot analysis. We observed that SMAD4 association with SMAD2 following TGF-β1 treatment was decreased upon HPV8 or MmuPV1 E6 co-transfection (Fig 4A). Similarly, formation of the SMAD3/SMAD4 complex was also inhibited upon HPV8 or MmuPV1 E6 expression (Fig 4B). These data support a model whereby HPV8 and MmuPV1 E6 inhibit TGF-β signaling by disrupting transcription factor complex formation at regulatory sites by excluding SMAD4 association with the receptor SMADs 2 and 3. Our results demonstrate that HPV8 E6 and MmuPV1 E6 can inhibit TGF-β signaling, including the induction of keratinocyte growth arrest and supports a model wherein E6 blocks the formation of DNA bound SMAD complexes by preventing SMAD4 from interacting with SMAD2/3.

### MAML1 binding defective MmuPV1 and HPV8 E6 mutants do not inhibit NOTCH signaling

**Fig 5:**
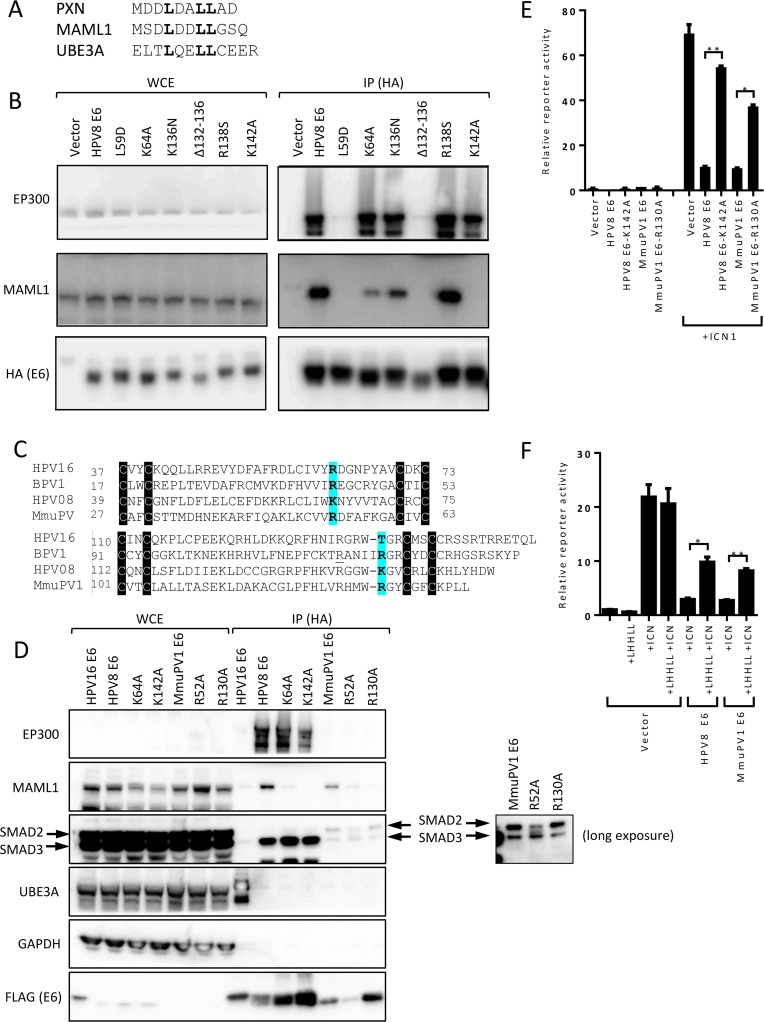
HPV8 and MmuPV1 E6 require interaction with MAML1 for NOTCH inhibition. **(A)** Alignment of LXXLL motifs of Paxillin, MAML1 and UBE3A. **(B) Lysates** of U2OS cells expressing FLAG/HA tagged versions of wildtype or mutant HPV8 E6 proteins were subjected to HA immunoprecipitation. Immunoprecipitated HPV8 E6 and associated EP300 and MAML1 were detected by immunoblotting. Expression levels of the various proteins were assessed by immunoblotting of whole cell extracts (WCE). GAPDH expression is shown as a loading control. **(C)** Alignment of amino acid sequences of HPV16, BPV1, HPV8 and MmuPV1 E6 proteins. E6 proteins contain two pairs of CXXC motifs that are highly conserved and essential for E6 structure and function[42], and therefore they are useful for aligning diverse E6 proteins. Shown are the regions between the first CXXC pair (top) and the start of the second paired CXXC through the rest of the sequence (bottom). **(D)** Lysates of U2OS cells expressing FLAG/HA tagged versions of wildtype or mutant HPV16, HPV8, and MmuPV1 E6 proteins were subjected to HA immunoprecipitation. Immunoprecipitated HPV8 E6 and associated EP300 and MAML1 were detected by immunoblotting. Interaction between HPV16 E6 and UBE3A is shown as a control. Expression levels of the various proteins were assessed by immunoblotting of whole cell extracts (WCE). GAPDH expression is shown as a loading control. **(E)** Effects of wild type and mutant HPV8 and MmuPV1 E6 on NOTCH responsive reporter activity using ICN1 as an agonist in U2OS cells. Data shown is from a representative experiment from three independent experiments **(F)** Effects of mutating the MAML1 LXXLL motif (MAML1-LHHLL) on HPV8 and MmuPV1 E6 mediated inhibition of an ICN1 activated NOTCH responsive luciferase reporter. Data shown is from a representative experiment from three independent experiments. P-values were calculated with unpaired t-test with Welch’s correction where * = P ≤ 0.05, ** = P ≤ 0.01.

The ability of HPV8 E6 to bind MAML1 has been suggested to be required for NOTCH inhibition and we predict that this interaction would also be required for MmuPV1 E6 inhibition of NOTCH signaling. We sought to directly test this hypothesis. HPV8 E6 interacts with an LXXLL motif in MAML1. Structural studies have identified the amino acid residues that make direct contact between BPV1 E6 and an LXXLL containing peptide derived from Paxillin (PXN) [42]. Based on these insights we set out to identify amino acid residues in HPV8 and MmuPV1 E6 that are necessary for MAML1 binding. In addition to PXN, BPV1 E6 can also interact with MAML1. There are key differences in the amino acid sequences surrounding the core LXXLL motifs of the HPV8 or MmuPV1 E6 binding PXN and MAML1, and UBE3A (E6AP) that binds to HPV16 E6 [42] but not to HPV8 or MmuPV1 E6 (Fig 5A). Therefore, we hypothesized that HPV8 and MmuPV1 E6 bind MAML1 using similar amino acid contacts as those required in BPV1 E6 for PXN binding. Hence we mutated putative LXXLL contact residues in HPV8 E6 that are conserved with BPV1 E6 and tested them for MAML1 binding. The following residues were targeted by mutation: leucine (L) 59, lysine (K) 64, arginine (R) 138, and K142. To test whether any of these mutations led to dramatic changes in E6 conformation, we measured binding to EP300, which does not contain an LXXLL motif. We used previously published EP300 binding defective mutants HPV8 E6 Δ132–136 and K136N mutants [37, 43] as controls and expected these to retain MAML1 binding. U2OS cells were transfected with plasmids expressing either wild type HPV8 E6 or HPV8 E6 mutants and analyzed E6 binding to endogenous EP300 and MAML1 by immunoblot. The HPV8 E6 L59D, K64A, and K142A as well as the EP300 binding deficient Δ132–136 and K136N mutants were all defective for MAML1 binding (Fig 5B). However, the L59D mutant was also defective for EP300 binding, similar to the previously described EP300 binding deficient Δ132–136 mutant; hence, these mutations likely result in global structural alterations. Contrary to what was previously published, the K136N mutant retained EP300 binding [37, 43]. The R138S mutant retained EP300 and MAML1 binding, whereas the K64A and K142A mutants were defective for MAML1 binding while retaining EP300 association.

Alignment of the HPV16, BPV1, HPV8, and MmuPV1 E6 protein sequences (Fig 5C), revealed that the positively charged K64 residue in HPV8 E6 corresponded to the positively charged R52 in MmuPV1 E6, and the positively charged K62 in HPV16 E6. In contrast, the positively charged K142 of HPV8 E6 corresponded to the positively charged R130 in MmuPV1, but corresponded to an uncharged T140 residue in HPV16. We mutated these residues in MmuPV1 E6 to create MmuPV1E6-R52A and MmuPV1E6-R130A and tested them for their ability to bind MAML1 and SMAD2/3 by immunoprecipitation followed by immunoblot (Fig 5D). Similar to the HPV8 E6 K64A and K142A mutants the MmuPV1 E6 R52A and R130A mutants showed diminished binding for MAML1 and retained the ability to bind to SMAD2/3. Given that the HPV8 E6 K64A and the MmuPV1 R52A mutants retained higher MAML1 binding than the HPV8 K142A and the MmuPV1 E6 R130A (Fig 5B and 5D) we chose the HPV8 K142A and MmuPV1 E6 R130A mutants for further analysis. The previously described NOTCH reporter assay was used to assess the ability of HPV8E6-K142A and the corresponding MmuPV1E6 R130A mutant to inhibit NOTCH signaling (Fig 5E). Consistent with decreased MAML1 association, these mutants were unable to fully inhibit NOTCH signaling. Lastly, to further confirm that NOTCH inhibition by MmuPV1 E6 was due to MAML1 binding, we mutated the two aspartate residues of the LDDLL motif in MAML1 to histidines (LHHLL). Using the NOTCH reporter assay we verified that the MAML1 LHHLL mutant was not dominant negative and that cotransfection of the MAML1 LHHLL mutant was able to partially block E6 mediated inhibition of NOTCH signaling (Fig 5F). Thus, either mutation of papillomavirus E6 residues that disrupt LXXLL binding or mutations in the MAML1 LXXLL motif interfere with E6 inhibition of NOTCH signaling. This shows that the ability of the HPV8 and MmuPV1 E6 proteins to inhibit NOTCH requires the interaction with MAML1 and does not occur through an indirect mechanism.

### HPV8 and MmuPV1 E6 inhibit differentiation and prolong survival of differentiated keratinocytes

**Fig 6:**
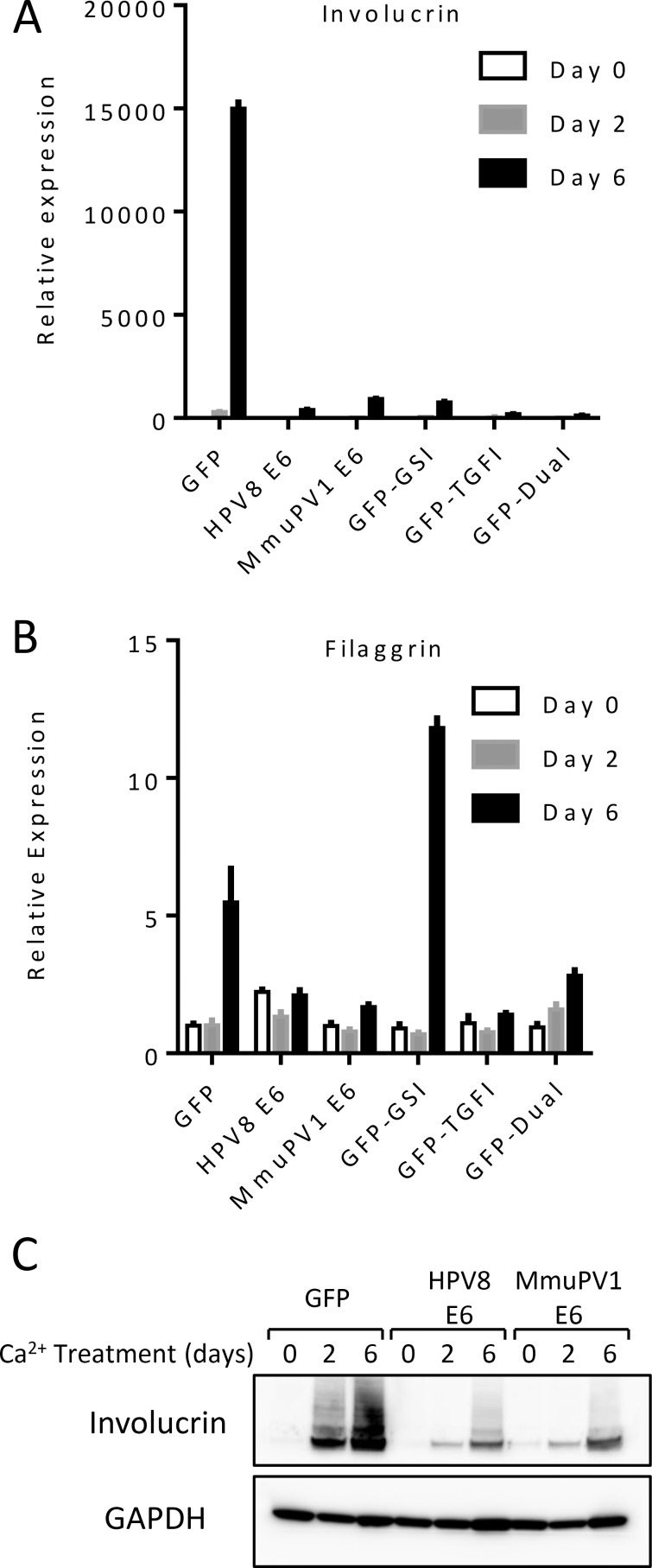
Expression of epithelial differentiation markers. GFP control telomerase immortalized oral keratinocytes (NOKs), control NOKs treated with GSI, TGFI, or a combination of both inhibitors, as well as HPV8 and MmuPV1 E6 expressing NOKs were differentiated in calcium containing media for six days and analyzed for expression of involucrin **(A)** and filaggrin **(B)** by RT-qPCR. The results of a representative experiment are shown with standard error of mean. Similar results were obtained in two additional experiments **(C)** Involucrin protein levels measured by immunoblot of NOKs during calcium induced differentiation.

After determining that MmuPV1 E6 and HPV8 E6 share the capacity to inhibit NOTCH and TGF-β signaling, we predicted that, similar to HPV8 E6 [18], MmuPV1 E6 can inhibit keratinocyte differentiation. NOTCH signaling is a critical driver of keratinocyte differentiation and negatively regulates proliferation [44]. Similarly, TGF-β has been implicated in maintaining epithelial stemness and controlling proliferation competency in differentiating keratinocytes [45]. We have previously shown that HPV8 E6 can inhibit differentiation of keratinocytes, a process at least partially dependent on NOTCH inhibition [18]. HPV8 and MmuPV1 E6 expressing telomerase immortalized oral keratinocytes (NOKs) were grown to confluency in low calcium containing, serum free medium and then switched to calcium containing DMEM supplemented with 10% FBS to induce differentiation for up to 6 days. RNA was isolated at days 0, 2, and 6, and expression of involucrin and filaggrin were measured by RT-qPCR. GFP expressing cells showed a marked increase in the levels of involucrin, a marker of intermediate stage keratinocyte differentiation, by day 6 (Fig 6A) which was absent from HPV8 E6 or MmuPV1 E6 expressing cells. Treatment of control keratinocytes with the gamma secretase inhibitor compound E (GSI), which blocks NOTCH cleavage, or the TGF-beta receptor 1 inhibitor SB-431542 (TGFI) either alone or in combination, similarly blocked the differentiation induced increase of involucrin mRNA. Next, we measured the expression of filaggrin, a marker of later stages of keratinocyte differentiation (Fig 6B). GFP expressing cells showed increased filaggrin expression after 6 days of calcium induced differentiation, but there was no similar increase in HPV8 E6 or in MmuPV1 E6 expressing cells. Treatment with TGFBI but not GSI blocked filaggrin expression. Filaggrin transcript levels were even higher during GSI treatment as compared to control cells, an observation that has also been seen in differentiated keratinocytes harboring heterozygous NOTCH1 deletions [46]. These authors suggested that NOTCH regulates intermediate differentiation and in the absence of signaling, differentiating keratinocytes prematurely initiate the late differentiation program including filaggrin expression. Additionally, we analyzed involucrin protein levels at days 0, 2, and 6 post-calcium induction by immunoblot. As observed in Meyers et al [18], control cells show robust induction of involucrin expression after 2 and 6 days of calcium treatment but this increase is not observed in HPV8 E6 and in MmuPV1 E6 expressing cells (Fig 6C).

**Fig 7:**
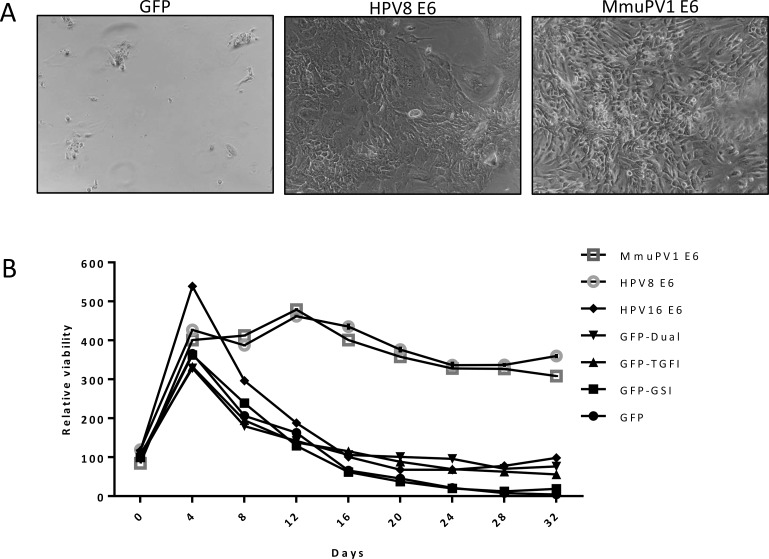
Survival of calcium differentiated keratinocytes. **(A)** Pictures of GFP-control (left), HPV8 E6 expressing (middle), and MmuPV1 E6 expressing (right) NOKs after 32 days in calcium differentiation medium. **(B)** Proliferation/viability of keratinocytes over 32 days of calcium differentiation measured by resazurin reduction. P-values (see text) were calculated using Wilcoxon ranked sum-test.

Calcium treatment of keratinocytes causes a decrease in proliferation and eventually death of terminally differentiated cells. We hypothesized that HPV8 and MmuPV1 E6 expressing cells would be resistant to these effects of terminal differentiation. GFP-control, HPV8 E6 or MmuPV1 E6 expressing NOKs were grown to confluency and switched to calcium-containing media. The medium was changed regularly, and the cells were observed for 32 days. Pictures were taken every two days until day 16 (S4 Fig) and at day 32 (Fig 7A). Before calcium addition, all cell populations had similar morphologies (S4 Fig). Most of the calcium treated control cells had expired and detached from the plate by day 32. In contrast, the HPV8 and MmuPV1 E6 expressing keratinocyte populations remained attached to the plate. In parallel we directly measured proliferation/viability of these cells using resazurin. Consistent with the observed changes in morphology, proliferation/viability of control cells started to decline after 4 days of calcium treatment and continued to decrease over the entire time period observed (Fig 7B). In contrast, HPV8 and MmupV1 E6 expressing keratinocytes compared to control cells remained metabolically active and survived throughout the 32 days of differentiation (p-values 0.0012 and 0.0028 respectively). Interestingly, however, chemical inhibition of NOTCH and/or TGF-β signaling in control keratinocytes or HPV16 E6 expressing keratinocytes did not significantly differ from control cells (p-values GSI 0.8203, TGFI 0.4363, dual 0.2973, HPV16 E6 0.2136). This indicates that HPV8 or MmuPV1 E6 expression prolongs the survival of differentiating keratinocytes, but that TGF-β and/or NOTCH inhibition does not account for these effects.

### MAML1 binding defective MmuPV1 E6 mutants do not cause papillomas in nude mice

**Fig 8:**
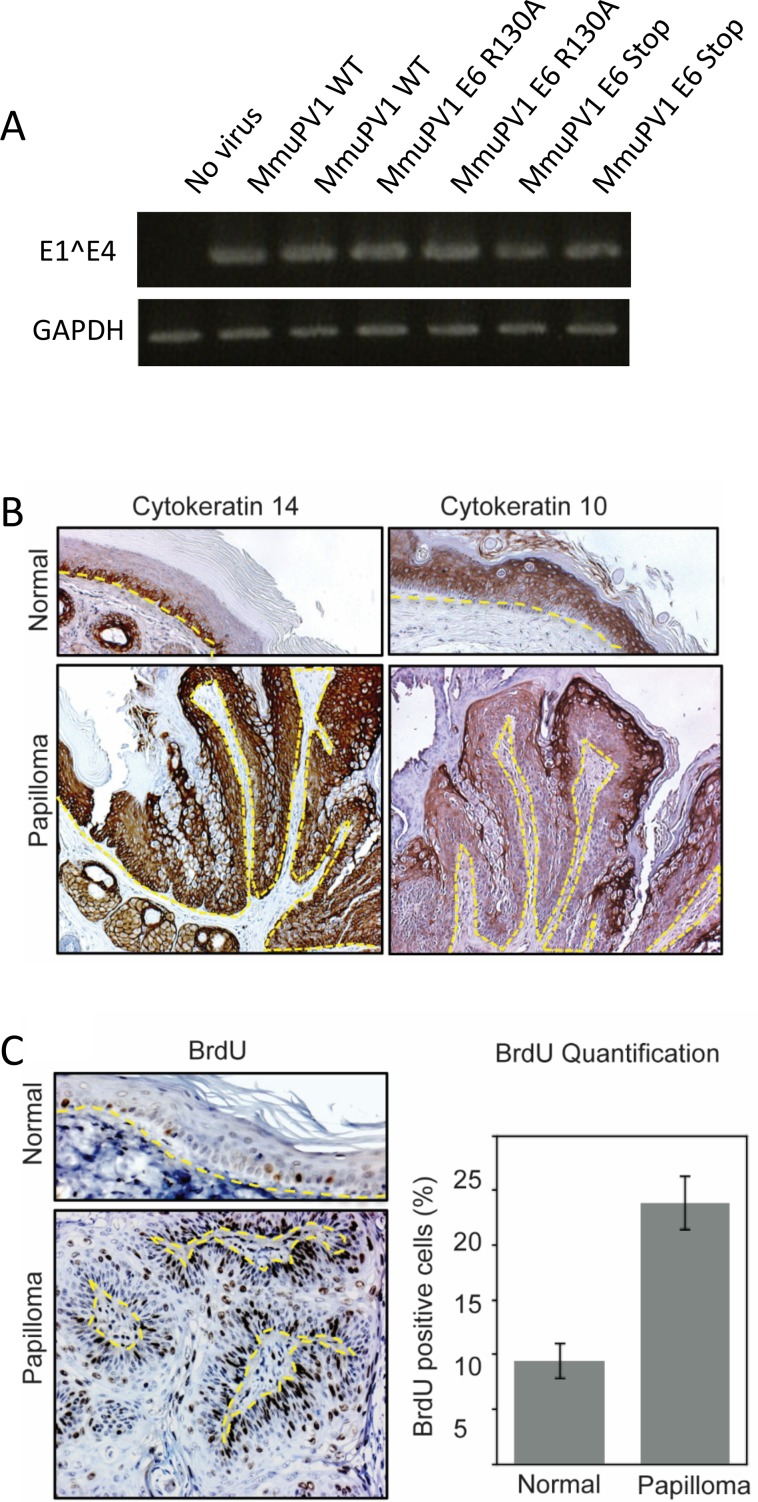
Infectivity of MmuPV1 quasivirus and histology and analysis of MmuPV1-induced papillomaviruses. **(A)** Quasiviruses were used to infect mouse keratinocytes and total RNA was harvested, cDNA was synthesized and assayed for the presence of E1^E4 spliced transcript. PCR products were visualized on agarose gel and compared to GAPDH amplification. **(B)** Immunohistochemistry analysis of differentiation markers in normal uninfected skin compared to MmuPV1 induced skin papillomas. Immunohistochemistry analysis of Cytokeratin14 (left), Cytokeratin10 (right) indicates delay in terminal differentiation program of epithelia in the papillomas. **(C)** Immunohistochemistry analysis of BrdU incorporation in cells of normal uninfected skin and papillomas. Quantification of the total percentage of BrdU-positive cells in papillomas (n = 3 mice) versus normal uninfected skin (n = 5 mice) from BALB/c-*Foxn1*^*nu*^ mice cells is shown in the bar graph (Error bars, SD. ** P < 0.05 using a two-sided Wilcoxon rank-sum test). The dotted lines indicate basement membrane of tissue.

MmuPV1 provides the ability to assess the biological importance of individual viral gene products and their biochemical activities to viral pathogenesis in vivo. To assess a role for E6 in virally induced pathogenesis, tails of 8–10 week old FoxN1^*nu/nu*^ mice were infected MmuPV1 quasivirions encapsulating wild type, E6-null (E6^STOP^) or E6^R130A^ mutant MmuPV1 genomes following topical scarification of the epidermis at the designated doses of virus (Table 1). Previous experiments indicated that infections with these doses of wild type viruses are sufficient to induce papillomas at 100% of sites infected [32]. Consistent with these findings, we found that infections with quasiviruses carrying wild-type MmuPV1 genomes induced papillomas at 100% efficiency. Unencapsidated, wild type MmuPV1 DNA is also infectious [31]. While 10 μg of wildtype MMuPV1 DNA induced warts at 100% of sites, the same amount of MmuPV1 E6^STOP^ or E6^R130A^ mutant DNA did not cause any papillomas (Table 2). To confirm that quasiviruses carrying E6^STOP^ or E6^R130A^ MmuPV1 mutant genomes were indeed infectious, we performed *in vitro* infections of mouse keratinocytes and tested for the transcription of E1^E4 spliced products by RT-PCR (Fig 8A). We found that E1^E4 spliced products could be detected post-infection with wild type as well as the mutant quasivirions, suggesting that mutant quasivirions are infectious but are defective for papilloma formation in immunodeficient mice.

**Table 1:** Papilloma incidence in FoxN1^*nu/nu*^ mice infected with MmuPV1 quasivirions.

Experiment No.	Genome	VGE	# Sites infected	# Sites w/ papilloma (%)
**1.**	MmuPV1-WT	2.48×10^7^	16	16 (100)
	MmuPV1-E6^Stop^	2.24×10^7^	16	0 (0)
	MmuPV1-E6^R130A^	0.98×10^6^	16	0 (0)
**2.**	MmuPV1-WT	2.11×10^8^	16	16 (100)
	MmuPV1-E6^Stop^	1.25×10^8^	16	0 (0)
	MmuPV1-E6^R130A^	2.26×10^8^	16	0 (0)

**Table 2:** Papilloma incidence in FoxN1^*nu/nu*^ mice infected with MmuPV1 DNA.

Genome	# Sites infected	# Sites w/ papilloma
MmuPV1-WT	16	16 (100)
MmuPV1-E6^Stop^	16	0 (0)
MmuPV1-E6^R130A^	16	0 (0)

### MmuPV1 induced papillomas show evidence of disrupted epithelial differentiation and increased cellular proliferation

Our previous *in vitro* experiments suggested that the ability of MmuPV1 E6 to inhibit NOTCH and TGF-β signaling would impair differentiation and increase cellular proliferation in MmuPV1-induced papillomas. Immunohistochemical analysis of differentiation layer markers cytokeratin 14 (K14) and cytokeratin 10 (K10) (Fig 8B) was used to assess the differentiation state of papillomas in comparison with normal uninfected skin. The papillomas showed expansion of basal-like epithelial cells as evidenced by immunohistochemical (IHC) analysis of the basal epithelial marker K14 (Fig 8B left panel). K10 expression is found in suprabasal compartment of normal, uninfected murine skin. MmuPV1-induced papillomas showed a delayed staining pattern of K10 in the papilloma (Fig 8B right panel)

To assess cellular proliferation in papillomas we evaluated incorporation of bromodeoxyuridine (BrdU) following intraperitoneal injection of the drug one hour prior to harvesting tissue. IHC was performed to identify BrdU-positive cells in normal epithelium or papillomas and the percentage of BrdU-positive cells was calculated. Approximately 9% of cells in normal uninfected tail skin of BALB/c-*Foxn1*^*nu*^ were BrdU-positive (Fig 8C), and positive cells were restricted to the basal layer of the epithelia. Due to the mildly hyperproliferative nature of nude mouse epithelia [47] the percentage of BrdU positivity is somewhat higher than in the skin of normal immunocompetent mice. Nevertheless, there was a significantly higher percentage of BrdU-positive cells (24%, p = 0.036) in the papillomas and BrdU positivity was not confined to basal cells (Fig 8C). This demonstrates that there is both an increase in cellular proliferation and licensing of DNA synthesis in suprabasal cells within MmuPV1-induced papillomas similar to what we observed in MmuPV1 E6 expressing iHFKs (Fig 2).

## Discussion

Cutaneous papillomaviruses such as HPV5 and HPV8 have long been associated with SCCs in EV patients and long-term immunosuppressed individuals [2, 7, 48]. Unlike cancers associated with mucosal HPV infections, cutaneous HPV associated SCC arise in sun exposed areas, and hence these viruses are thought to cooperate with UV to induce SCCs [49]. HPV8 E2, E6 and E7 each have oncogenic activities when expressed in basal epithelial cells of transgenic mice, but the molecular mechanisms by which these HPVs contribute to cancer development have remained enigmatic. Moreover, HPV sequences are not maintained in every cancer cell, and hence cutaneous HPVs are not necessary for the maintenance of the transformed state [10].

Given the well documented interaction with UV exposure, most molecular studies with cutaneous HPVs have focused on modulation of cell cycle arrest, apoptosis and DNA repair after UV irradiation [50]. HPV8 E6 has been reported to interact with the pro-apoptotic BCL2 family member BAK1 thereby inhibiting apoptosis in response to UV [51]. In addition, cutaneous HPV E6 proteins have been reported to inhibit ATM and ATR activation through an EP300 dependent mechanism, thereby inhibiting DNA repair in response to UV exposure [16, 38]. Interactions with BAK and EP300 have also been reported for mucosal HPV E6 proteins [14, 52–54].

Several studies, however, have identified cellular proteins that specifically interact with cutaneous HPV5 and HPV8 E6 but not mucosal HPV E6 proteins. These include members of receptor regulated SMADs (R-SMADs) 2 and 3, which are key to TGF-β signaling [23, 33, 35]. In addition, HPV5 and 8 E6 bind to MAML1, an essential co-activator of NOTCH signaling [19, 20, 35]. HPV8 and MmuPV1 E6 proteins inhibit these pathways through stoichiometric interactions and they do not appear to destabilize these proteins by co-opting the cellular ubiquitin conjugation machinery. While mucosal HPV E6 proteins do not detectably interact with MAML1 or SMAD2 and SMAD3, they may target these pathways through other mechanisms. High-risk HPV E6 proteins may inhibit some aspects of NOTCH signaling indirectly by targeting the TP53 tumor suppressor for degradation [55] and high-risk HPV16 E7 can inhibit TGF-β mediated growth inhibition [56] and interact with SMAD3 [57, 58]. Even though TGF-β and NOTCH are important tumor suppressor pathways in keratinocytes, it is unclear whether and how inhibition of these pathways by cutaneous HPV E6 proteins contribute to induction of lesions and cancer.

The lack of an animal system where the biological relevance of specific viral host interactions can be investigated with respect to the viral life cycle and pathogenesis has greatly hindered papillomavirus research. While early studies with bovine papillomavirus 1 (BPV1) and cottontail rabbit papillomavirus 1 (CRPV1; more recently referred to as *Sylvilagus floridanus* Papillomavirus 1—SfPV1) enabled infections of autologous hosts, the respective host animals are not genetically tractable. The isolation of MmuPV1 from cutaneous warts of immunodeficient nude mice and the fact that it can be used to experimentally infect laboratory mice have been important steps towards enabling viral pathogenesis studies. Moreover, the recent discovery that MmuPV1-induced warts can undergo malignant progression when subjected to UV [32] suggests that experimental MmuPV1 infections may allow modeling some aspects of SCC formation by cutaneous HPVs.

As a first step towards determining whether MmuPV1 may be a useful pathogenesis model of cutaneous HPV infections, we investigated whether MmuPV1 E6 shared cellular interactors with HPV5 and HPV8 E6 proteins. We initially focused on MAML1, the R-SMADs SMAD2 and SMAD3 that are required for TGF-β signaling and EP300. We found that similar to what has been reported for HPV5 and 8 E6 [23, 33, 35], MmuPV1 interacts with MAML1 and TGF-β R-SMADs (Fig 1A), thereby inhibiting these two important tumor suppressor pathways (Fig 1B and 1C). Our results suggest that similar to what we previously reported for HPV8 E6, MmuPV1 E6 inhibits NOTCH by interacting with a nuclear transcription factor complex that contains MAML1 and cleaved, active intracellular NOTCH (ICN) [18]. HPV8 and MmuPV1 E6 also share the ability to associate with TGF-β R-SMADs, but HPV8 E6 appears to preferentially associate with SMAD3, whereas MmuPV1 E6 associates preferentially with SMAD2 (Fig 1A). This was somewhat surprising given the high degree of sequence identity (83.9%) between the two proteins. However, in both cases, E6/R-SMAD associations inhibit transcriptional responses to TGF-β (Fig 1B). Our results do not support earlier studies that reported SMAD3 destabilization in HPV5 E6 expressing cells [23] (Fig 1A and Fig 3A). Moreover, HPV8 and MmuPV1 E6 do not appear to markedly affect R-SMAD phosphorylation and nuclear translocation (Fig 3A). Our results (Figs 3B and 4) suggest a model whereby E6 R-SMAD binding inhibits SMAD4 binding and thus formation of an active transcriptional complex. Cancer-associated SMAD2 mutations are also defective for SMAD2/SMAD4 complex formation [59], and cutaneous papillomavirus E6 proteins seem to functionally mimic SMAD2 mutations. Since MmuPV1 and HPV8 E6 can be detected at the SRE of the CDKN2B promoter (Fig 3B), we propose that similar to what has been observed in their inhibition of NOTCH signaling, these E6 proteins interfere with the transcriptional activity of a DNA bound SMAD2/SMAD3 containing transcription factor complex. Additional experiments are required, however, to carefully test this model. SMAD2 and SMAD3 do not contain recognizable LXXLL motifs and hence they are expected to bind different E6 sequences than the LXXLL containing MAML protein. Consistent with that notion, we found that the MAML binding defective HPV8 and MmuPV1 E6 mutants retained SMAD2/3 binding (Fig 5D).

In contrast to HPV8 E6, MmuPV1 E6 proteins does not detectably bind EP300 (Fig 1A and Fig 5D). We also tested MmuPV1 E6 EP300 binding in murine cells and did not detect an association. Similar to SMAD2 and SMAD3, EP300 does not have an LXXLL motif. EP300 is an important co-activator for many different transcriptional programs including NOTCH signaling. EP300 associates with a C-terminal sequence of MAML1 that is referred to as Transcriptional Activation Domain (TAD)1 [60], whereas MmuPV1 and HPV8 E8 associate with a separate LXXLL motif containing domain referred to as TAD2 and hence do not directly compete for EP300 binding to TAD1. Based on our finding that MmuPV1 does not co-precipitate EP300 and that MAML1 defective HPV8 E6 mutants retain EP300 association we conclude that EP300 binding to HPV8 E6 is not mediated through MAML1 and moreover, that E6 binding to TAD2 may prevent EP300 binding to TAD1. This may provide a molecular mechanism for inhibition of NOTCH transcription by cutaneous papillomavirus E6 proteins. In addition, given that MmuPV1 associated warts can progress to SCCs, it would appear that EP300 binding is not strictly required to cooperate with UV for SCC formation. Many studies that have implicated EP300 as a major cellular effector of cutaneous HPV E6 activities have been based upon the use of the HPV8 E6 Δ132–136 and K136N mutants [37, 43]. Our experiments unexpectedly revealed that the Δ132–136 mutant is also defective for MAML1 binding, whereas the K136N mutant did not exhibit any overt defects for EP300 binding (Fig 5B). Caution should be taken when interpreting data obtained with these mutants.

The NOTCH and TGF-β tumor suppressors are critical determinants of differentiation and cell fate in keratinocytes and act by coordinating cell-cycle withdrawal and driving keratinocytes toward terminal differentiation and ultimately, cell death [61, 62]. Our results show that similar to MmuPV1 induced cutaneous warts, HPV8 and MmuPV1 E6 expressing human keratinocytes are differentiation resistant and remain proliferatively active (Figs 6 and 7). The differentiation process is largely TGF-β and NOTCH dependent as TGF-β and/or NOTCH inhibitor treatment of normal keratinocytes mimics the effects of E6 expression, but extended survival of differentiated keratinocyte was independent of these two pathways (Fig 7B). In addition, we also observed that HPV8 and MmuPV1 E6 expressing keratinocytes remain viable for extended periods of time under conditions that induce differentiation (Fig 7). Interestingly, TGF-β and/or NOTCH inhibition in normal keratinocytes was not sufficient for this phenotype (Fig 7B).

Taken together, our results suggest HPV8 and MmuPV1 E6 allow infected cells to remain proliferatively active and not only resist differentiation cues but also remain viable over extended periods of time. This would be manifested by an expansion of basal-like, proliferatively active as is seen in MmuPV1 induced skin lesions (Fig 8).

The number of cutaneous HPVs that have been isolated and characterized has dramatically increased over the last few years. In addition to HPV5 and HPV8, a large number of beta genus HPVs and more recently also gamma genus HPVs have been detected in cutaneous lesions and SCCs [10, 63]. Proteomic studies of E6 associated cellular proteins have started to shed some light on similarities and differences of cellular pathways that may be targeted these by the various cutaneous HPVs [33].

The next important challenge will be to determine how subversion of these various pathways contributes to the pathogenesis and oncogenicity of these viruses. Are there low-risk and high-risk cutaneous HPVs? If so, is the oncogenic potential dependent on inhibition of specific cellular pathways as has been shown for mucosal HPVs?

Our results provide evidence that MmuPV1 will be an important, biologically relevant model to address some of these issues, particularly as they relate to NOTCH and TGF-β inhibition by E6. Our experiments show that MmuPV1 E6 expression is necessary for wart formation and that a MmuPV1 quasivirus carrying a genome encoding a MAML1 binding defective E6 mutant does not cause wart formation (Tables a and b). Using recently published structures of papillomavirus E6 proteins bound to cellular targets we may be able to generate MmuPV1 mutants that are defective for binding to R-SMADs or other associated cellular target proteins and test these both *in vitro* and *in vivo*.

Even without such an E6 mutant we can perform additional experiments with small molecule inhibitors and/or by infecting mouse strains, even immune competent mice [32], that carry mutations in specific signaling pathways to conclusively assess the importance of E6 mediated NOTCH and TGF-β inhibition for MmuPV1 replication and pathogenesis *in vivo*.

## Materials and Methods

### Cell culture

U2OS human osteosarcoma and HCT116 human colon carcinoma cells were obtained from ATCC and grown in Dulbecco’s Modified Eagle Medium (DMEM) with high glucose (Gibco) supplemented with penicillin-streptomycin and 10% fetal bovine serum (FBS). Tert-immortalized human foreskin keratinocytes Cl398 [64] (iHFKs) (obtained from Al Klingelhutz, University of Iowa) or normal oral keratinocytes (NOK) [65] were maintained in keratinocyte serum free media (KSFM) (Gibco) supplemented with 0.2 ng/ml EGF, 25mg/ml bovine pituitary extract, and penicillin-streptomycin. Cells were differentiated by switching KSFM to DMEM/10% FBS. Recombinant human TGF-β1 (Millipore) was used at a final concentration of 10 ng/ml in all experiments. Compound E (Millipore) and SB-431542 (Sigma) were used at 2 μM and 10 μM respectively.

### Plasmids, transfections, and lentiviral transduction

Plasmids used in transient transfections were pCMV BamNeo vectors with Flag-hemagglutinin epitopes fused to the amino termini of HPV E6 proteins: pNCMV (vector) HPV16 E6, HPV8 E6, MmuPV1 E6. Lentiviral plasmids used were generated through cloning of pLenti6.3 /V5 TOPO Gateway compatible vector (Invitrogen): GFP (control), HPV8 E6, HPV16 E6, and MmuPV1 E6. Notch reporter construct, HES1-luc[66], HA-tagged ICN1[67], and MAML1 full-length[67] expression plasmids (obtained from Jon Aster, Harvard Medical School) were used as previously described [68]. TGF-β reporter construct, (CAGA)_9_-MLP-Luc (obtained from Jennifer Pietenpol, Vanderbilt University School of Medicine) was used as previously described [69]. SMAD2 and SMAD3 expression plasmids were obtained from Michael Hoffman (University of Wisconsin). All mutations were created using QuikChange II site-directed mutagenesis kit (Agilent). Transient transfections of U2OS cells was performed using Polyethylenimine (PEI) (Polysciences) as described [70] and analysis of transfected cells was performed at 48 hours post-transfection. Transient iHFK transfections were performed using Fugene 6 (Promega) and were analyzed at 48 hours post transfection. Preparation of and infection with recombinant lentiviruses was as previously described [71]. Selection of infected cells using Blasticidin (10 μg/ml) began two days post infection and was maintained for seven days. Cells were than maintained as described in KSFM.

### Luciferase reporter assays

Reporter assays were performed using Dual-Luciferase Reporter Assays System (Promega). Lysates of cells transfected with the appropriate plasmids (200 ng reporter, 200 ng vector or E6, 10 ng renilla, and 200 ng ICN1 or 200 ng MAML1 where appropriate) were prepared in 100 μl of passive lysis buffer at 48 hours after transfection and 20 μl of lysate was used for each reading. Readings were done in triplicate using a LMax II plate reader (Molecular Devices) and values normalized for transfection efficiency using the co-transfected renilla luciferase expression plasmid.

### RNA isolation and real-time quantitative PCR analysis

RNA was isolated using Quick-RNA MiniPrep (Zymo Research). cDNA was synthesized using Quantitect Reverse Transcription Kit (Qiagen). Quantitative PCR (qPCR) was performed in triplicate on a StepOne Plus (Applied Biosystems) thermocycler using SYBR Green PCR Master Mix (Applied Biosystems) reagents. PCR primers used are listed in S1 Table. Data shown was calculated using ΔΔCT method and normalized to expression of the RPLP0 as the housekeeping gene.

### Immunoprecipitations, fractionation and immunoblotting

Cells were lysed in 1% NP40 buffer (1% Nonidet P-40 (NP40), 120mM NaCl and 50mM TrisHCl (pH 8.0). Immunoprecipitations of HA epitope tagged proteins were performed using HA antibodies coupled to agarose beads (Sigma). Samples were run on NuPAGE 4–12% Bis-Tris Gels (Invitrogen) according to manufacturer’s instructions. Proteins were electrotransferred to Polyvinylidene fluoride (PVDF) membranes (Immobilon-P; Millipore). The membranes were blocked in 5% nonfat dry milk in TBST (137 mM NaCl, 2.7 mM KCl, 25 mM Tris [pH 7.4], 0.1% Tween 20) and probed with the appropriate antibody. Primary antibodies (1:1000 dilution) used for immunoblots are listed in S2 Table. Secondary anti-mouse and anti-rabbit antibodies conjugated to horseradish peroxidase (Amersham) were used at dilutions of 1:10,000. Subcellular fractionation was done using the REAP method [72]. In brief, cell pellets were washed with phosphate buffered saline (PBS) and repelleted. The cytoplasmic fraction was isolated by tituration of the pellet in PBS containing 0.1% NP40. The nuclear pellet was washed and resuspended in 1% NP40 lysis buffer followed by sonication and treatment with Pierce Universal Nuclease (Thermo Fisher Scientific).

### Resazurin-based viability/proliferation assay

Resazurin was used at 25 μg/ml in PBS to assess redox fitness [73]. Cells were incubated with dye for one hour and then sample fluorescence was read in triplicate using 560 nm excitation and 590 nm emission filters on a Synergy H1 microplate reader (BioTek).

### Chromatin Immunoprecipitation

Chromatin was prepared using SimpleChIP Enzymatic Chromatin IP Kit (Cell Signaling Technology) according to manufacturer’s instructions and approximately 10^6^ cells were used for each assay. Antibodies used are listed in S2 Table. Co-precipitated DNA was isolated according to manufacturer’s protocol and quantitative PCR was performed as described previously [18]. Data is expressed as compared to percent input after subtraction of isotype matched IgG signal.

### Animals

Immunodeficient euthymic BALB/c FoxN1nu/nu (Harlan) were used in this study. All infected mice were housed in aseptic conditions in micro-isolator cages. Animals were handled only by designated personnel and personal protection gear was changed between cages to prevent any cross contamination from virus.

### Ethics statement

All animal experiments were performed in full compliance with standards outlined in the "Guide for the Care and Use of Laboratory Animals” by the Institute of Laboratory Animal Resources (ILARC) of the Commission on Life Sciences (CLS), National Research Council (NRC) as specified by the Animal Welfare Act (AWA), associated Animal Welfare Regulations (AWRs), Public Health Service (PHS) Policy and Office of Laboratory Animal Welfare (OLAW) and approved by the Governing Board of the National Research Council (NRC), whose members are drawn from the councils of the National Academy of Sciences (NAS), National Academy of Engineering (NAE), and Institute of Medicine (IM). Mice were housed at McArdle Laboratory Animal Care Unit in strict accordance with guidelines approved by the Association for Assessment of Laboratory Animal Care (AALAC), at the University of Wisconsin Medical School. All protocols for animal work were approved by the University of Wisconsin Medical School Institutional Animal Care and Use Committee (IACUC, Protocol number: M02478).

### Infection of nude mice with MmuPV1 wild type or mutant quasivirions

Infections were performed using quasivirions containing MmuPV1 wild type or mutant genomes as described previously [74, 75]. Briefly, 293FT cells (ATCC) were cotransfected with a MmuPV1 capsid protein expression plasmid (pMusSheLL- a gift from Chris Buck, National Cancer Institute) [30, 76] and MmuPV1 wild type or mutant DNA for encapsidation. After 48 h at 37°C, cells were harvested and virions were purified using Optiprep gradient centrifugation. The generated quasivirions were quantified and used to infect of FoxN1^nu/nu^ mice (Harlan) as described [32]. Briefly, *in vivo* infections with purified MmuPV1 quasivirions were performed on scarified skin of the animals’ tails. Animals were anesthetized and four spots on tails were scarified using a 27-gauge syringe needle to scrape the epithelia (not sufficient to cause bleeding) followed by pipette delivery of virus solution using a siliconized pipette tip.

### Infection of nude mice with wild type or mutant MmuPV1 genomes

*In vivo* infection with wild type or mutant MmuPV1 genomes was as previously published reports [30, 31, 76, 77] with some modifications. The viral genomes were recovered by excision from the plasmid backbone using the restriction enzyme XbaI, followed by intramolecular religation using T4 DNA ligase, as detailed on the website of the Laboratory of Cellular Oncology (http://home.ccr.cancer.gov/Lco). Animals were scarified as described above and four days post-scarification inoculated with 10 μg recircularized viral DNA (in a 10 μl volume) by injecting with a 30-gauge needle into the scab.

### Detection of MmuPV1 E1^E4 spliced transcripts

Mouse keratinocytes JB6-clone 41 (gift from Dr. Nancy H. Colburn, NCI) were maintained in modified Eagle’s Medium MEM containing 5% FBS and infected with MmuPV1 mutant or wild-type quasivirions at a multiplicity of infection of 10. Forty-eight hours post-infection, total RNA was isolated using the RNeasy kit (Qiagen) and reverse-transcribed into cDNA using SuperScript III (LifeTechnologies) as per manufacturers’ instructions. E1^E4 transcripts were analyzed by PCR. GAPDH was used as a positive control. Primer sequences for the detection of MmuPV1 E1^E4 spliced transcripts and GAPDH have been published previously [76] and are listed in S1 Table. PCR products were resolved by Agarose gel electrophoresis.

### Tissue procurement and histochemical analysis

Skin was harvested, fixed in 4% paraformaldehyde, and embedded in paraffin. Serial sections (5 μm thick) were analyzed for Keratin markers and BrdU. For immunohistochemistry, sections were deparaffinized and rehydrated with xylenes and graded ethanol, respectively. Endogenous peroxidase activity was quenched with 3% H_2_O_2_ in methanol and followed with heat-induced antigen retrieval in 10 mM citrate, pH 6.0. Antigen antibody complexes were detected with biotinylated horse anti-mouse/rabbit IgG (Vector Laboratories) and were visualized with 3,3′-diaminobenzidine (Vector Laboratories). Tissues were counterstained with hematoxylin. All images were taken with a Zeiss AxioImager M2 microscope using the AxioVision software version 4.8.2.

### Bromodeoxyuridine incorporation

To assess cellular proliferation, we evaluated incorporation of bromodeoxyuridine (BrdU) (203806, Calbiochem) at one hour after intraperitoneal injection. Tissue was harvested and processed for immunohistochemistry using a BrdU antibody as described above. For each experimental group (normal skin and papillomas), three slides, each derived from an individual animal, were analyzed by microscopy. Ten random fields of normal skin or the papilloma were selected on each slide and the total number of epithelial cells and the number of BrdU-positive cells were manually counted. The percentage of BrdU-positive cells was calculated. A two-sided Wilcoxon rank-sum test was used to compare the average percentage of BrdU-positive cells between the two groups.

## Supporting Information

S1 Fig Inhibitory effects of tagged and untagged versions of HPV8 and MmuPV1 E6. Effects of N-terminally tagged and untagged HPV8 E6 and MmuPV1 E6 on TGF-beta and NOTCH reporter activity in U2OS cells.
(TIF)

S2 Fig Inhibitory effects of HPV8 E6 and MmuPV1 E6 in iHFKs. Panel (A) shows activity of SMAD responsive promoter when induced by the constitutively active receptor TGFBR1 T204D. Panel (B) shows activity of the NOTCH responsive promoter when induced by ICN.
(TIF)

S3 Fig Characterization of HPV8 E6 and MmuPV1 E6 interactors. WCE of iHFKs expressing GFP, HPV8 E6, or MmuPV1 E6 were immunoprecipitated with HA antibody beads and analyzed for association with p300 SMAD2, SMAD3, pSMAD2, pSMAD3, and SMAD4.
(TIF)

S4 Fig Calcium differentiation of immortalized keratinocytes. NOK cells expressing GFP, HPV8 E6, or MmuPV1 E6 were differentiated in calcium for 16 days and pictures were obtained every two days.
(PDF)

S1 Table List of primers used in this study. (DOCX)

S2 Table List of antibodies used in this study. (DOCX)
