# Supplementary figures and images for: Cutaneous HPV8 and MmuPV1 E6 Proteins Target the NOTCH and TGF-β Tumor Suppressors to Inhibit Differentiation and Sustain Keratinocyte Proliferation
Source: PLoS Pathog. 2017 Jan 20;13(1):e1006171. doi: 10.1371/journal.ppat.1006171 (PMC5287491; doi:10.1371/journal.ppat.1006171)

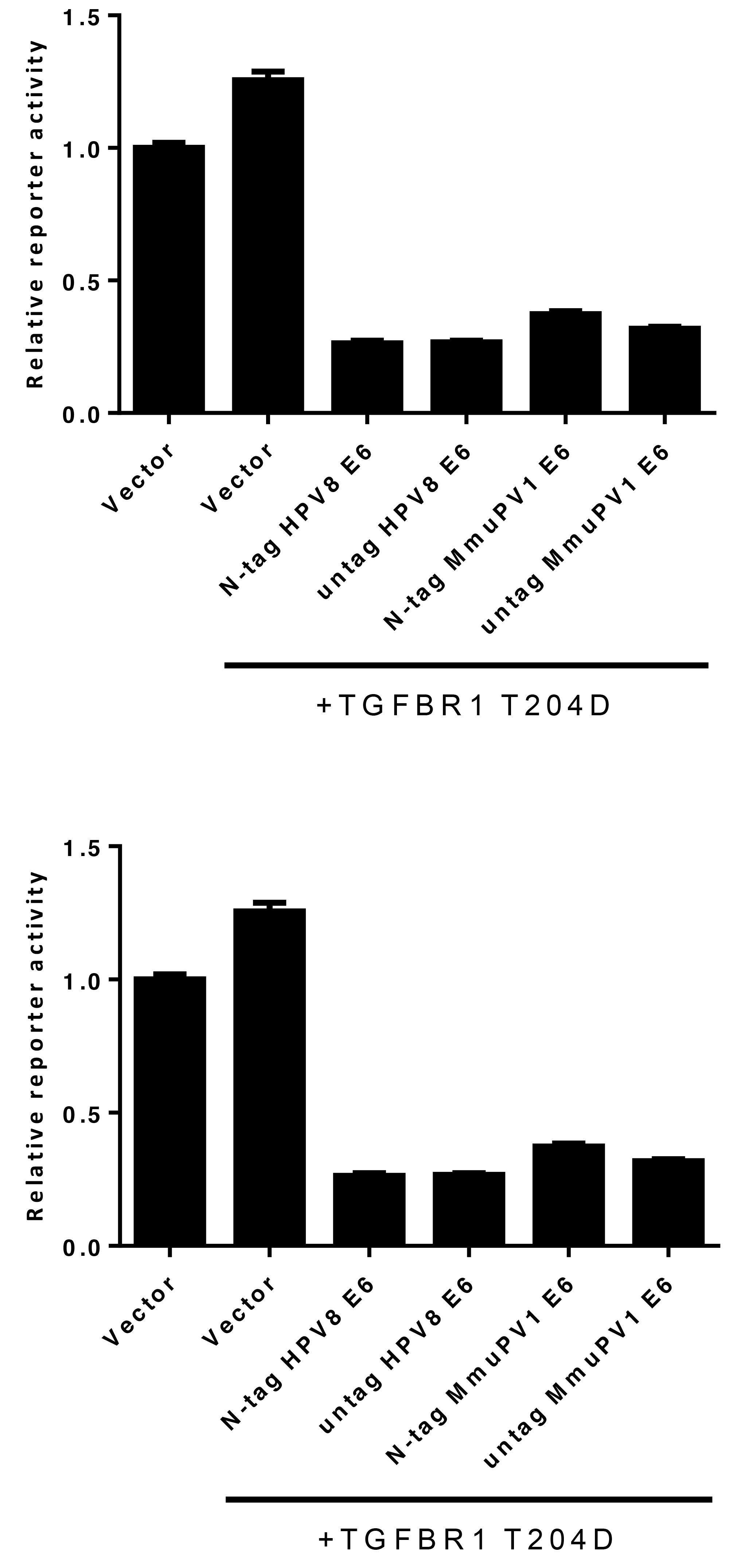

Supplement: S1 Fig — Effects of N-terminally tagged and untagged HPV8 E6 and MmuPV1 E6 on TGF-beta and NOTCH reporter activity in U2OS cells. (TIF) [file ppat.1006171.s001.tif]

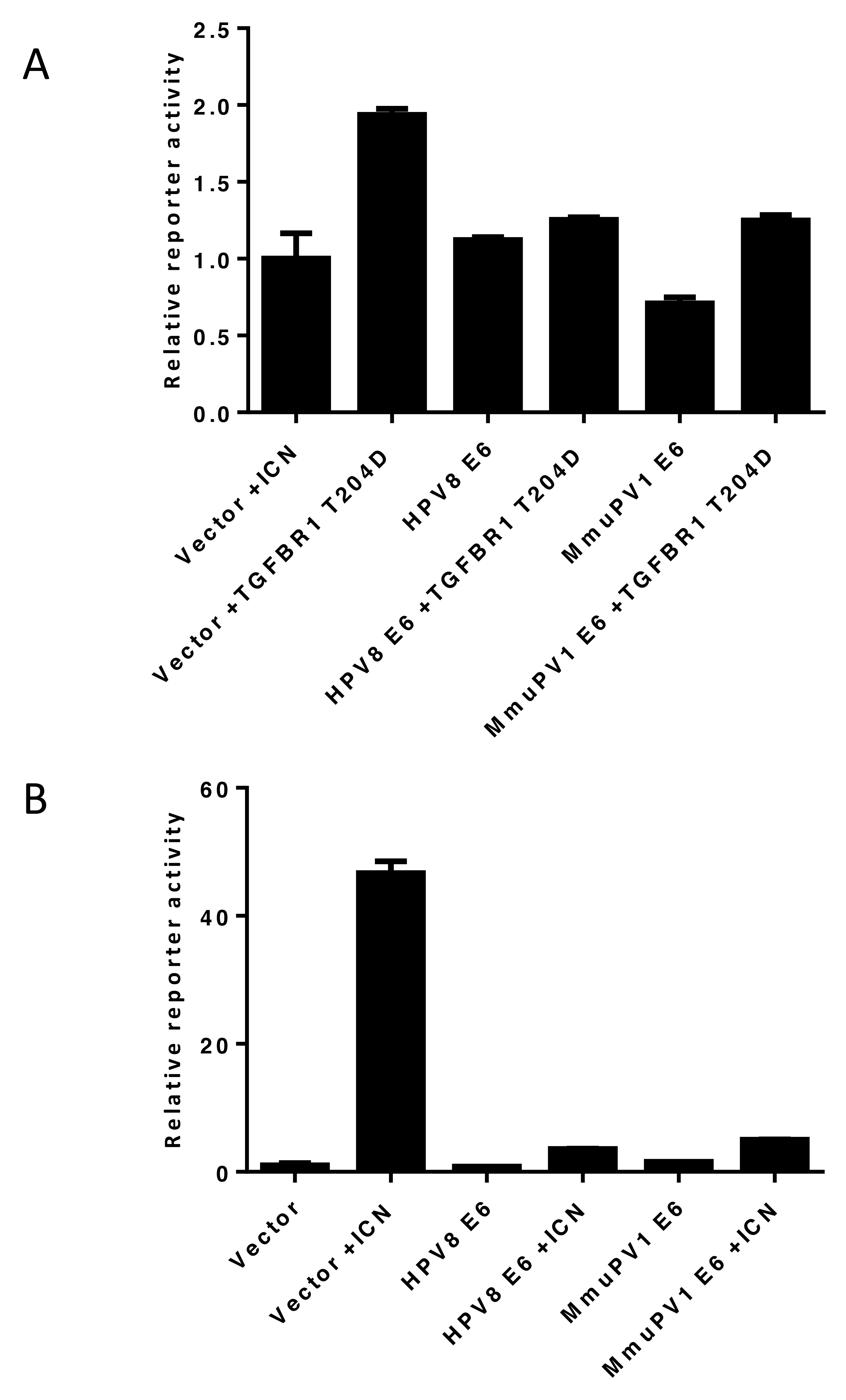

Supplement: S2 Fig — Panel (A) shows activity of SMAD responsive promoter when induced by the constitutively active receptor TGFBR1 T204D. Panel (B) shows activity of the NOTCH responsive promoter when induced by ICN. (TIF) [file ppat.1006171.s002.tif]

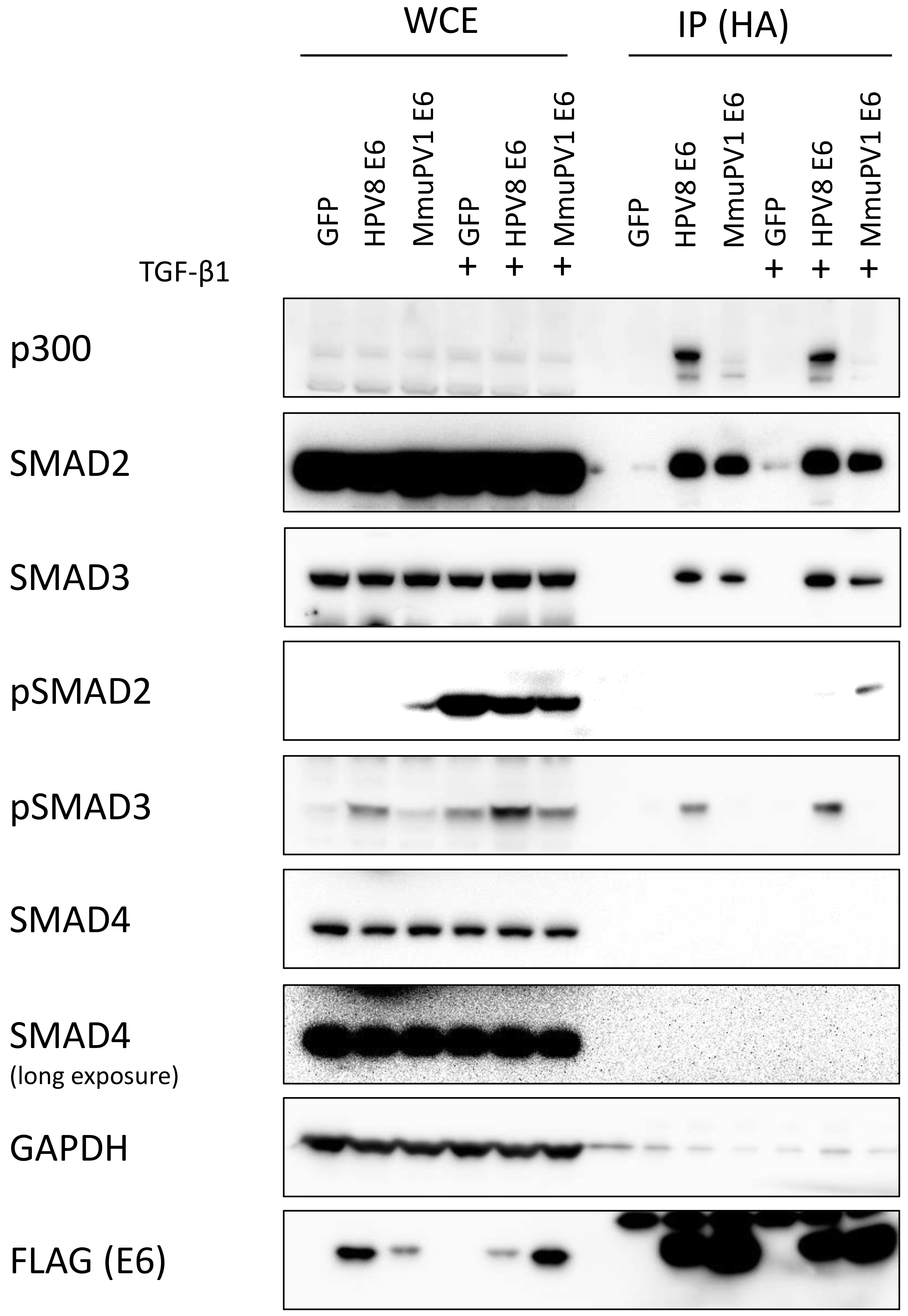

Supplement: S3 Fig — WCE of iHFKs expressing GFP, HPV8 E6, or MmuPV1 E6 were immunoprecipitated with HA antibody beads and analyzed for association with p300 SMAD2, SMAD3, pSMAD2, pSMAD3, and SMAD4. (TIF) [file ppat.1006171.s003.tif]

## GFP NOKs

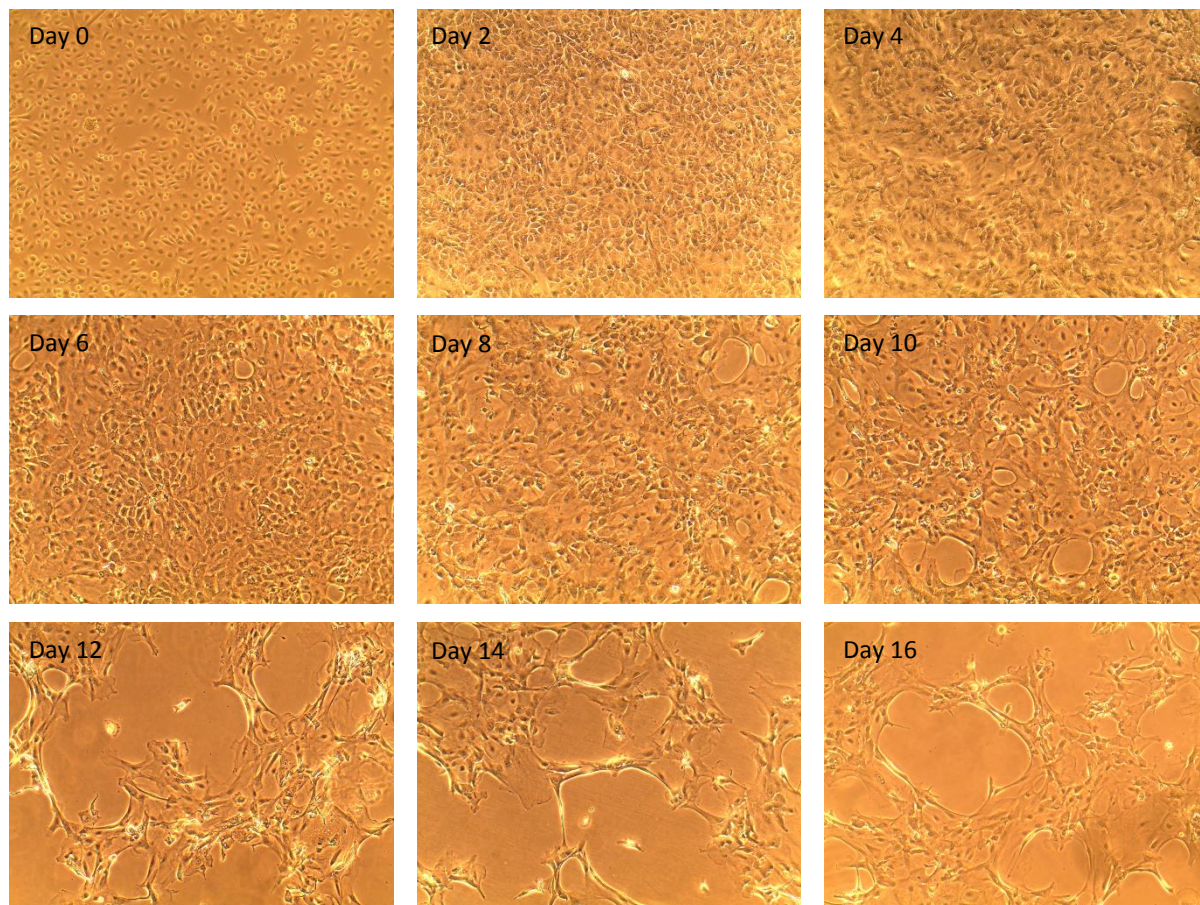

Supplemental Figure 4

## HPV8 E6 NOKS

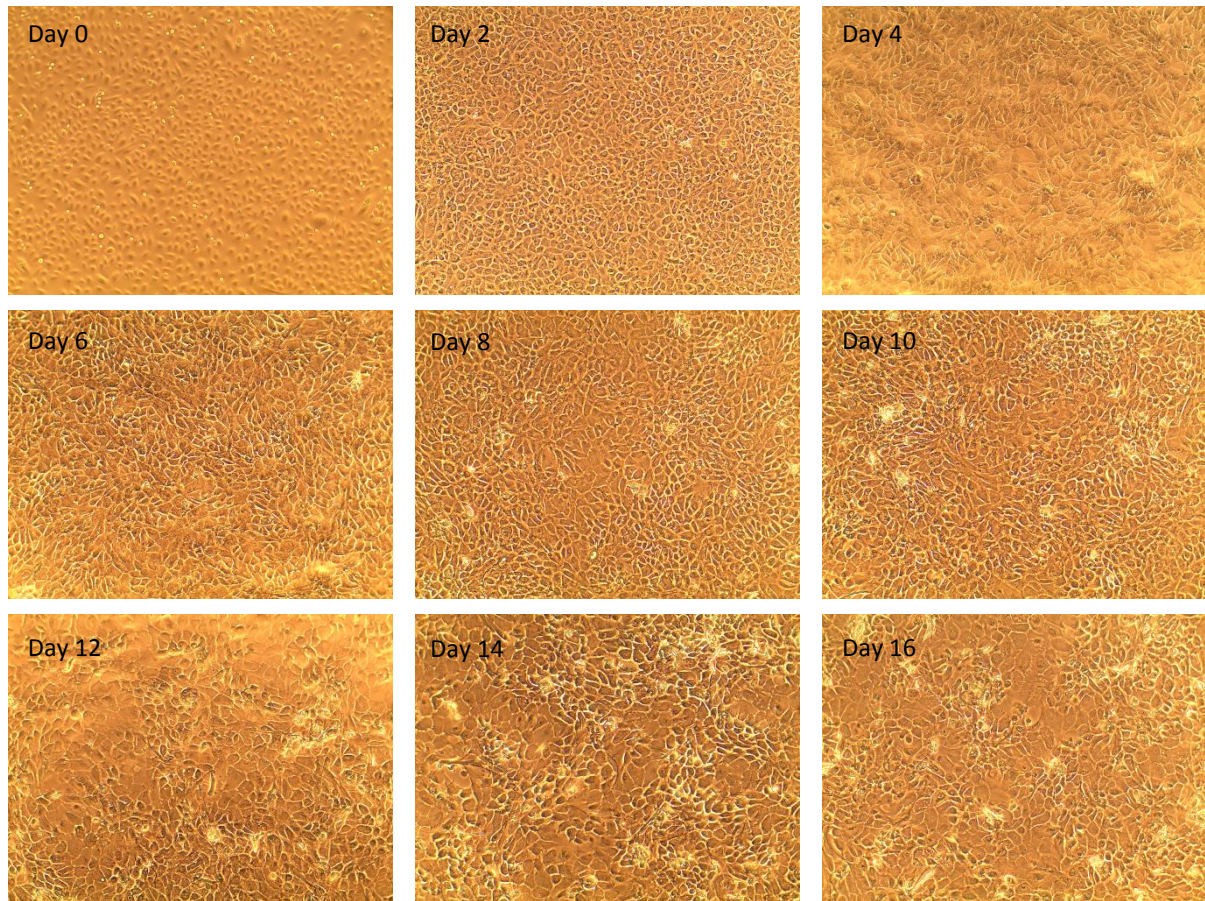

Supplemental Figure 4

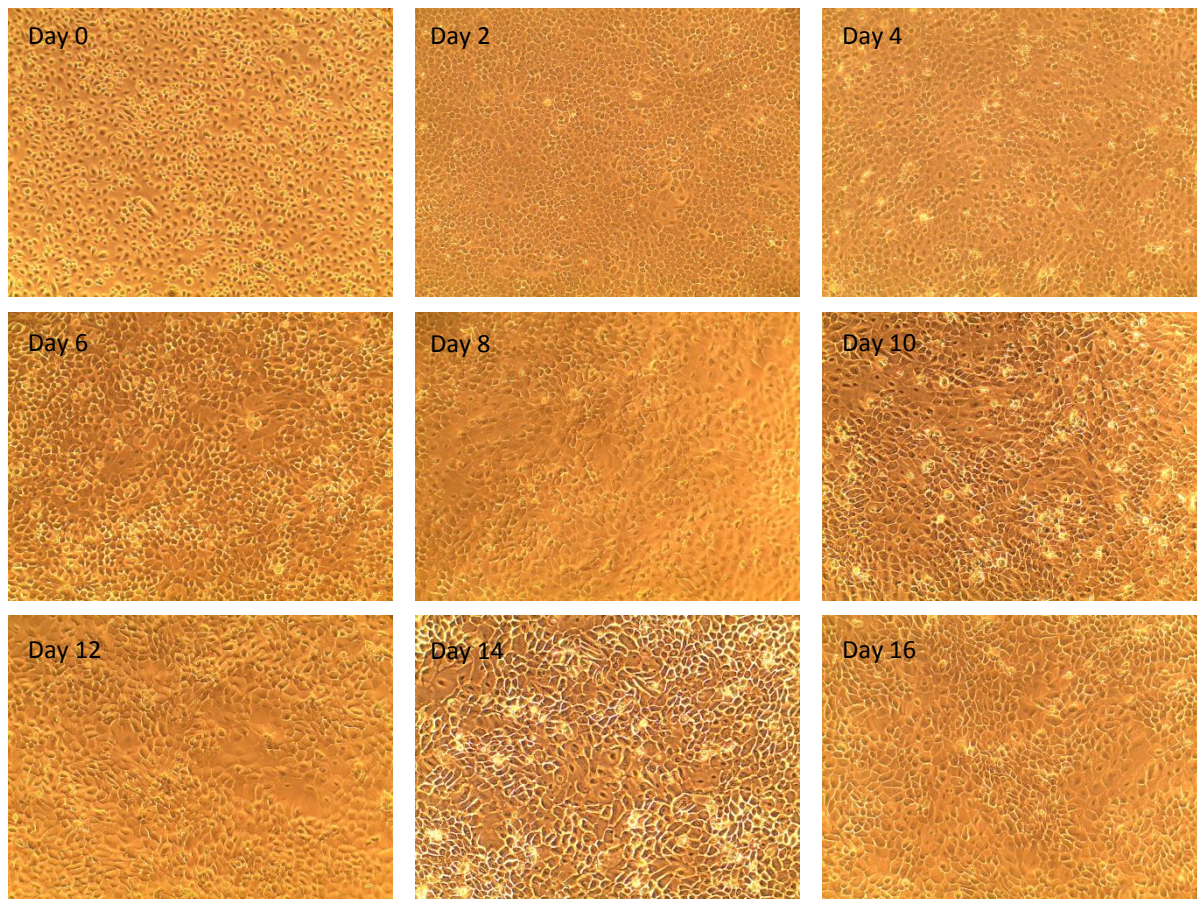

Supplemental Figure 4

Supplement: S4 Fig — NOK cells expressing GFP, HPV8 E6, or MmuPV1 E6 were differentiated in calcium for 16 days and pictures were obtained every two days. (PDF) [file ppat.1006171.s004.pdf]
