# Supplementary material for: Cutaneous HPV8 and MmuPV1 E6 Proteins Target the NOTCH and TGF-β Tumor Suppressors to Inhibit Differentiation and Sustain Keratinocyte Proliferation
Source: PLoS Pathog. 2017 Jan 20;13(1):e1006171. doi: 10.1371/journal.ppat.1006171 (PMC5287491; doi:10.1371/journal.ppat.1006171)
Supplement: S1 Table — (DOCX) [file ppat.1006171.s005.docx]

**S1 Table. List of primers used in this study**

QPCR primers

Involucrin Forward 5’ TGC CTG AGC AAG AAT GTG AG -3’

Involucrin Reverse 5’- TGC TCT GGG TTT TCT GCT TT -3’

Filaggrin Forward 5’- AAA GAG CTG AAG GAA CTT CTG -3’

Filaggrin Reverse 5’- AAC CAT ATC TGG GTC ATC TGG -3’

HES1 Forward 5’-GGA AAT GAC AGT GAA GCA CCT CC-3’

HES1 Reverse 5’-GAA GCG GGT CAC CTC GTT CAT G-3’

CDKN2B Forward 5’- AGA TCC CAA CGC CCT GAA -3’

CDKN2B Reverse 5’- CCC ATC ATC ATG ACC TGG ATT -3’

RPLP0 Forward 5’- ATC AAC GGG TAC AAA CGA GTC -3’

RPLP0 Reverse 5’- CAG ATG GAT CAG CCA AGA AGG -3’

E1^E4 Forward, 5′- CAT TCG AGT CAC TGC TTC TGC -3′

E1^E4 Reverse, 5′- GAT GCA GGT TTG TCG TTC TCC -3′

GAPDH Forward, 5’- ACC ACA GTC CAT GCC ATC AC -3’

GAPDH Reverse, 5’- TCC ACC ACC CTG TTG CTG TA -3’

ChIP primers

CDKN2B promoter Forward 5’- CAT GAT TCT CGG GAT TTT TCT C -3’

CDKN2B promoter Reverse 5’- GCG ACA GCT CTG CAC C -3’
