# Supplementary material for: Cutaneous HPV8 and MmuPV1 E6 Proteins Target the NOTCH and TGF-β Tumor Suppressors to Inhibit Differentiation and Sustain Keratinocyte Proliferation
Source: PLoS Pathog. 2017 Jan 20;13(1):e1006171. doi: 10.1371/journal.ppat.1006171 (PMC5287491; doi:10.1371/journal.ppat.1006171)
Supplement: S2 Table — (DOCX) [file ppat.1006171.s006.docx]

**S2 Table. List of antibodies used in this study**

EP300, clone RW128 (Millipore 05-257)

MAML1, D3K7B (Cell Signaling Technology 12166)

Cleaved Notch1 (ICN1), Val1744, D3B8 (Cell Signaling Technology 4147)

SMAD2, D43B4 (Cell Signaling Technology 5339)

SMAD3, C67H9 (Cell Signaling Technology 9523)

SMAD2/3, D7G7 (Cell Signaling Technology 8685)

pSMAD2, 138D4 (Cell Signaling Technology 3108)

pSMAD3, C25A9 (Cell Signaling Technology 9520)

SMAD4 for ChIP (Cell Signaling Technology 9515)

SMAD4, 10HCLC (ThermoFisher 710714)

HA for ChIP, C29F4 (Cell Signaling Technology 3724)

HA, Y-11 (Santa Cruz Biotech sc-805)

FLAG, M2 (Sigma F1804)

GAPDH, G9 (Santa Cruz Biotech sc-365062)

Involucrin, SY5 (Santa Cruz Biotech sc-21748)

CDKN1A, F-5 (Santa Cruz Biotech sc-6246)

IgG for ChIP (Cell Signaling Technology 2729)

Histone H3 for ChIP, D2B12 (Cell Signaling Technology 4620)

Histone H2A, D603A (Cell Signaling Technology 12349)

BrdU (Oncogene NA20)

Keratin K10 (Covance PRB-159P)

Keratin K14 (Covance PRB-155P)
